# Prioritizing Risks and Uncertainties from Intentional Release of Selected Category A Pathogens

**DOI:** 10.1371/journal.pone.0032732

**Published:** 2012-03-06

**Authors:** Tao Hong, Patrick L. Gurian, Yin Huang, Charles N. Haas

**Affiliations:** 1 National Exposure Research Laboratory, Environmental Protection Agency, Athens, Georgia, United States of America; 2 Department of Civil, Architectural, and Environmental Engineering, Drexel University, Philadelphia, Pennsylvania, United States of America; 3 Office of Biostatistics and Epidemiology, Food and Drug Administration, Rockville, Maryland, United States of America; Tulane School of Public Health and Tropical Medicine, United States of America

## Abstract

This paper synthesizes available information on five Category A pathogens (*Bacillus anthracis*, *Yersinia pestis*, *Francisella tularensis*, *Variola major* and Lassa) to develop quantitative guidelines for how environmental pathogen concentrations may be related to human health risk in an indoor environment. An integrated model of environmental transport and human health exposure to biological pathogens is constructed which 1) includes the effects of environmental attenuation, 2) considers fomite contact exposure as well as inhalational exposure, and 3) includes an uncertainty analysis to identify key input uncertainties, which may inform future research directions. The findings provide a framework for developing the many different environmental standards that are needed for making risk-informed response decisions, such as when prophylactic antibiotics should be distributed, and whether or not a contaminated area should be cleaned up. The approach is based on the assumption of uniform mixing in environmental compartments and is thus applicable to areas sufficiently removed in time and space from the initial release that mixing has produced relatively uniform concentrations. Results indicate that when pathogens are released into the air, risk from inhalation is the main component of the overall risk, while risk from ingestion (dermal contact for *B. anthracis*) is the main component of the overall risk when pathogens are present on surfaces. Concentrations sampled from untracked floor, walls and the filter of heating ventilation and air conditioning (HVAC) system are proposed as indicators of previous exposure risk, while samples taken from touched surfaces are proposed as indicators of future risk if the building is reoccupied. A Monte Carlo uncertainty analysis is conducted and input-output correlations used to identify important parameter uncertainties. An approach is proposed for integrating these quantitative assessments of parameter uncertainty with broader, qualitative considerations to identify future research priorities.

## Introduction

Biological weapons, also known as “the poor man's atom bomb”, have been included in terrorists' arsenal because of their capability of producing mass causalities combined with natural access to the pathogens, manageable technical challenges and relatively low costs to launch an attack [1], [2], [3]. Prior to the 2001 anthrax letter attacks, identified bioterrorism attacks included the release of *Salmonella typhimurium* to eleven restaurant salad bars in the city of Portland in 1984 to influence an election, which caused the infection of 750 people, and the release of *B. anthracis* spores in Tokyo by the religious group Aum Shinrikyo between 1990 and 1995, which failed to infect any people [4], [5]. The 2001 anthrax letter attacks infected 22 people (11 inhalational cases and 11 cutaneous cases [6]), caused the deaths of 5 people, and cost hundreds of millions of dollars in clean up costs [7]. The attacks revealed that the U.S. lacked the guidelines for a quick response to such attacks, as well as decontamination standards for bioterrorism agents [8].

As a result, research has been undertaken to better understand the risks resulting from a bioterrorist attack. Sextro et al. modeled the spread of *B. anthracis* spores in a hypothetical office suite, estimated occupants' exposure, and found that activity-related resuspension was an important source of human exposure [9]. This model did not consider environmental decay of the pathogen. While *B. anthracis* is a persistent pathogen whose environmental decay rate can be treated as zero for a short time simulation [10], Sextro et al.'s model would need to be modified to include environmental attenuation in order to be used to estimate the fate and transport of non-persistent biological agents. Price et al. [11] created a framework to link the degree of contamination in a building to the risk to the occupants, which could also be used to establish a decontamination standard if an acceptable risk level is provided. In addition, Price et al. linked the number of negative samples to the level of statistical confidence in the determination that the building had been effectively decontaminated [11]. However, this study did not provide a mechanistic model to describe the long term fate and transport and overall mass balance of the released pathogens, instead using a proportionality relationship to link the short term surface concentration of deposited pathogens to the short term concentration of aerosolized ones. Hong et al. [12] modeled the distribution of both air and surface-released *B. anthracis* spores in an office, and used concentrations found in different environmental media (i.e., surface, wall, ventilation filter, etc.) to infer future or past aerosol exposure. At the same time, they applied probability sampling theory in determining the minimum sampling area corresponding to certain levels of confidence in meeting allowable residual risk targets. The variability during sampling recovery and the potential for clumping of *B. anthracis* were taken into account. Besides not including pathogen decay, the above-mentioned studies quantify only inhalational risk, and omit threats from ingestion and dermal contact.

While models for *B. anthracis* have focused on a single pathway, inhalation exposure, mathematical models have been developed for influenza that take multiple disease transmission routes, such as inhalation and ingestion, into account [13], [14], [15]. Nicas and Gang introduced a Markov chain model to quantify multiple-pathway exposure to influenza for a health-care worker who had close contact with a patient [16]. Three exposure routes were concerned, hand-mucous membranes, inhalation, and direct projection of pathogen-containing droplets onto mucous membranes. In a subsequent study, Nicas et al. applied their model to quantify the relative importance of different influenza virus exposure pathways, and pointed out that model uncertainties had significant impacts on the conclusion as to which pathway is dominant [17]. Atkinson and Wein constructed a four-person household transmission model to quantify the dominant transmission route for pandemic influenza [14], [15]. Both of the studies performed analysis on the recognized major transmission pathways: droplet, airborne, and contacts [18], [19]. However, the above-mentioned studies adopted fixed parameter values in the computations instead of distributions across possible values, which does not account for variability and uncertainty. There is evidence that including uncertainty and variability is important [20], [21]. Smieszek compared predictions from a mechanistic exposure model and empirical data from a contact diary study to analyze the impacts of different contact intensities and durations. Results showed that treating all the contacts equally overestimated the expected number of infected individuals [20]. A study by Julian et al. used Monte Carlo simulation to analyze variability and uncertainty in the risk due to nondietary ingestion of rotavirus, relying on a micro-level activity time series [21], which may inspire future high-resolution microbial risk assessment [22].

These multiple pathway models have been applied to common transmissible pathogens but have not addressed Category A agents. They have generally sought to identify which pathways are of concern, rather than informing the development of quantitative standards for response actions. To address the need for such quantitative standards this paper synthesizes available information on five Category A pathogens to develop a framework for relating environmental pathogen concentrations to human health risk. The five pathogens considered are: *B. anthracis*, *Y. Pestis*, *F. tularensis*, *Variola major*, and Lassa. Properties of each of these pathogens are described below.

*B. anthracis* is a Gram-positive, facultatively anaerobic, rod-shaped bacterium of the genus *Bacillus*. It is the causative agent of anthrax, an acute disease in humans and animals, which is highly lethal in some forms. *B. anthracis* is one of only a few bacteria that can form long-lived spores. *Y. pestis*, the causative agent of plague, is a Gram-negative facultative anaerobic bipolar-staining bacillus bacterium belonging to the family *Enterobacteriaceae*. Plague may be manifested in one of three forms: bubonic, pneumonic, and septicemic plague [23]. *Francisella tularensis* is a pathogenic species of Gram-negative bacteria that causes the zoonotic disease tularemia. *F. tularensis* is reported to be one of most infectious organisms known. It is an intracellular pathogen, replicating mainly in macrophages, and has also been reported in amoebae [24]. *Variola major* is the causative agent of smallpox. There has been no effective treatment developed for this disease, which has an average 30% mortality rate. Lassa virus, the causative agent of one type of hemorrhagic fever, infects more than 200,000 people per year causing more than 3,000 deaths with a mortality rate of about 15% among the hospitalized cases [25]. The selected Category A pathogens represent a range of environmental persistencies from a pathogen with a very low decay rate (*B.anthracis*), to several with high decay rates (*Y.Pestis*, *F. tularensis*, and Lassa), as well as one with a moderate decay rate (*Variola major*).

The objective of this study is to expand the framework that Hong et al. [12] developed for linking environmental concentrations of *B. anthracis* with human health risk by 1) including the effects of environmental attenuation, 2) considering a variety of different pathogens instead of a single one (*B. anthracis*), 3) taking account of contact exposure (ingestion or dermal risk) as well as inhalational exposure, and 4) conducting an uncertainty analysis and identifying key input uncertainties. Both detailed and reduced form solutions to the equations linking risk to environmental concentrations are developed, which could benefit in making risk-informed response decisions, such as determining when prophylactic antibiotics should be distributed, and whether or not a contaminated area should be cleaned up. Monte Carlo methods are used to assess uncertainty in the results and identify important uncertainties in input parameters so that future research may be directed towards reducing them.

## Methods

### 2.1 Fate and transport model

**Figure 1:**
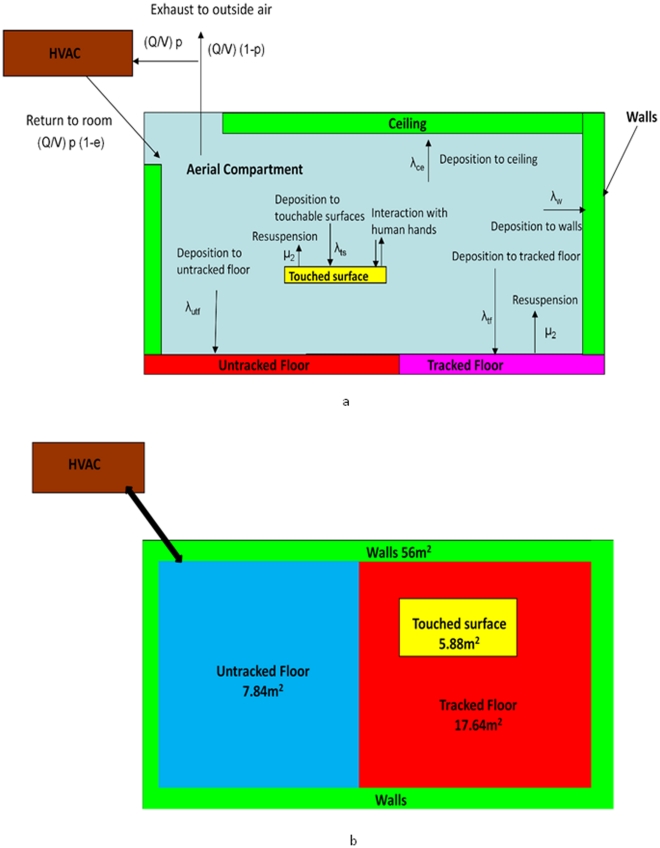
Schematic of model. (HVAC stands for heating ventilation and air conditioning. a. cross section view, b. plan view).

In this study, an occupant is modeled as continuously present in a one-room office with a heating, ventilation, and air conditioning (HVAC) system (Figure 1). This person has the chance of inhaling aerosolized pathogens and ingesting pathogens deposited on the touched surfaces through the surface-hand-mouth transmission route. For *B. anthracis*, the ingestion risk is replaced by cutaneous risk since this was a more important exposure route than ingestion in the 2001 anthrax letter attacks [6]. The room is modeled as a set of completely mixed compartments. This assumption fails to capture localized areas of high risks, such as a high concentrated puff of pathogens right after the initial release. Thus, the approach developed here is more appropriate for situations somewhat removed in time and space from the initial release, where mixing has occurred and concentrations are relatively uniform.

The governing equation for the fate and transport of released pathogens is presented in Equation 1:
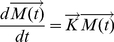
(1)and its initial conditions (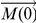) is defined as:
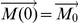



The general solution to Equation 1 is expressed as:
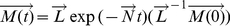
(2)where 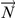 and 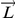 contain the eigenvalues and eigenvectors after eigendecompositing the transfer matrix 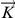 based on Equation 3 [26]:
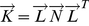
(3)


For the fate and transport model used in this paper, 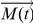 is a vector representing the numbers of pathogens in each of 10 modeled states with subscripts: 1) air (indoor air, M_air_), 2) ts (horizontal touchable surfaces from which spores may be transferred to human hands, M_ts_), 3) tf (tracked floor from which spores may be re-suspended by walking or other activities, M_tf_), 4) utf (untracked floor from which there is no re-suspension, M_utf_), 5) w (walls, M_w_), 6) f (HVAC filter, M_f_), 7) n (the nasal passages, M_n_), 8) h (hands of an occupant of the office, M_h_), 9) ec (all areas external to the room, M_ec_), and 10) d (decayed pathogens,M_d_). Thus Equation 1 can be detailed written as:
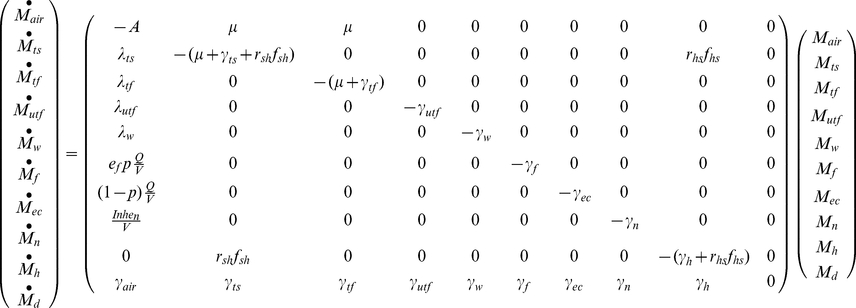
(4)where
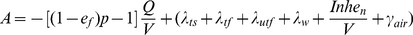
(5)


The solution to this set of ordinary differential equations gives the concentration of pathogens in the 10 different compartments as a function of time. Because the system is linear, masses in different compartments will be proportional to the release quantity. Thus, solving the system gives the constant of proportionality so that measured surface concentrations can be related to release quantity and risk to occupants. In Equation 4, the deposition from the air compartment to compartment i is modeled as a first-order process with rate constants of λ_i_ (with i values as described above). A second source of removal is by the HVAC system. The total air flow rate through the HVAC system is denoted by Q (units of m^3^/s), p (dimensionless) is the fraction of total air flow that is recirculated into the building by the HVAC system, e_f_ (dimensionless) is the efficiency of the filter at removing particles, and V is the volume of the room (m^3^). Removal to the occupants' nasal passages is also modeled with Inh (m^3^/s), denoting the breathing flow rate, and e_n_ (dimensionless), the efficiency of the nasal passages at removing particles. Removal by losing viability in each compartment is modeled as a first order rate with separate decay rates, γ_i_. However, due to limited data, only two types of decay values are available (Table 1), 1) the air decay rate (γ_air_), which is used for the air and external compartments, and 2) the fomite decay rate (γ_fomite_), which is used for the loss of viability in other media. Resuspension from the tracked floor due to occupants walking and other activities is also modeled as a first order process with rate constant μ (units of s^−1^). The interactions between human and fomites are represented by hand-surface (r_hs_) and surface- hand (r_sh_) contact rates, as well as mass transfer fractions between hand to surface (f_hs_), surface to hand (f_sh_), and hand to mouth (f_hm_).

**Table 1:** Category A Pathogen's Environmental Persistency.

Pathogen	Averaged decay rate in the air (γ_air_) (hr^−1^)	Range of decay rate in the air (γ_air_) (hr^−1^)^a^	Condition	Source	Averaged decay rate on fomite (γ_fomite_) (hr^−1^)	Range of decay rate on the fomite (γ_fomite_) (hr^−1^)^a^	Condition	Source
*B. anthracis*	8.16×10^−5^	(1.11×10^−5^, 1.97×10^−4^)	NA	[43], [44]	3.36×10^−5^	(1.92×10^−5^, 4.64×10^−5^)	NA	[43], [45], [46], [47]
*Y. pestis*	2.75	(2.10, 3.49)	T = 26°C, rH = 20–87%	[48]	4.55×10^−1^	(0.04, 1.24)	T = 11–22°C, rH = 30–55% metal, steel, glass, paper, and Polyethylene	[49], [50]
*F. tularensis*	3.27	(0.55, 9.20)	T = 20–40°C, rH = 85%	[51], [52], [53]	2.39×10^−1^	(0.01, 0.46)	T = 25–37°C, rH = 10–100% on metal	[50]
*Variola major*	4.55×10^−2^	(1.00×10^−2^, 1.30×10^−1^)	T = 10–34°C, rH = 20–80%	[54], [55]	6.89×10^−3^	(5.45×10^−3^, 9.95×10^−3^)	T = 25–37°C, rH = 3–96% on glass	[56]
Lassa	2.6	(0.78, 4.14)	T = 24–28°C, rH = 30–80%	[57]	7.67×10^−1^ b	(0.68, 0.92)	T = 20°C, rH = NA on aluminum	[58]

a Uniform distribution is assumed between the maximum and minimum values.

b Due to the lack of information on Lassa, the average of the decay rates of *Bunyaviridae hantavirus*, Sicilian virus Sabin, and Crimean-Congp on fomites are used for Lassa.

The deposition rates can be expressed in terms of parameters representing the indoor air flow conditions [27], [28], [29]:
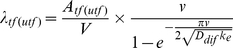
(6)

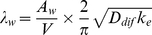
(7)

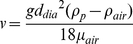
(8)where A_tf(utf)_ is the surface area of the tracked or untracked floor, A_w_ is the surface area of the walls, D_dif_ is the particle's diffusivity, k_e_ is turbulence intensity, *v* is particle settling velocity, which is given in Equation 8 as a function of the gravitational constant (g), the particle's diameter (d_dia_), the viscosity of air (μ_air_), the density of the particle (ρ_p_), and the density of air (ρ_air_).

### 2.2 Release scenarios

Two pathogen release scenarios are considered. In the first scenario, pathogens are released to the air compartment. The occupant directly inhales aerosolized pathogens and ingests the deposited ones via surface-hand-mouth contacts. Environmental concentrations measured at the end of the exposure period are used to characterize the risk from the past aerosol release, and as such it is termed the retrospective scenario. In the second scenario, pathogens are initially present on the touched surfaces, where they may be ingested by surface-hand-mouth contacts. In addition, human-caused resuspension introduces the pathogens into the air where they can be inhaled by the occupant. Environmental concentrations at the beginning of the exposure period are used to predict the future risk and as such the scenario is termed the prospective scenario. This scenario addresses the residual risk present after aerosolized particles have had the opportunity to deposit onto surfaces, a key issue in establishing a decontamination standard.

The exposure dose (d_dose_) is composed of two sources: inhalation and ingestion. Based on Equation 9, the inhalation dose is obtained by integrating the inhalation rate (Inh) and the pathogen's air concentration (C_air_) over the total exposure duration (the exposure begins at t_1_ and ends at t_2_, while the agent is introduced at t = 0), and the ingestion dose equals the integral of the hand-mouth contact rate (r_hm_), mass transfer fraction from hand to month during each contact (f_hm_), the involved area of a human hand (A_h_), and the pathogen's concentration on the hand (C_h_) over the same exposure period:
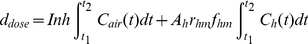
(9)For dermal contact, ingestion dose is replaced by the total amount of pathogen transferred to the hand from touched surfaces:
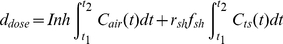
(10)


This equation is applied for *B. anthracis* instead of Equation 9, where C_ts_ is pathogen's concentration on the touched surface. However, separate dose-response coefficients are used for the different exposure pathways for anthrax. The dermal dose-response parameter is tuned so as to produce equal numbers of dermal and inhalation cases for the aerosol release scenario, as was observed in the 2001 attacks.

### 2.3 Dose-response functions

The exponential (Equation 11) and beta-Poisson (Equation 12) dose-response models, which have been widely used in microbial risk assessment [30], are used in this study:
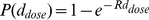
(11)

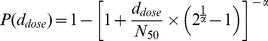
(12)


In Equations 11 and 12, *P*(d_dose_) is the probability of positive response (infection, illness, or death) for a population average dose, which allows for Poisson variability in individual exposure [31]. R is the parameter of the exponential dose-response model, *N*_50_, and *α* are the parameters of the beta-Poisson model. When the risk is relatively small, a first-order Taylor series expansion can be used to approximate Equations 11 and 12 as [12]:
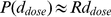
(11a)

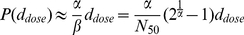
(12a)


This transformation can simplify the low dose risk estimation, which is where this approach is intended for use (i.e., for areas removed from the initial release where concentrations will be relatively uniform over spatial scales of interest. When the exposure dose is high, the full model (Equations 11 and 12) should be used.

### 2.4 Linking pathogen concentrations to risk

#### 2.4.1 Retrospective scenario

Given that it is rarely possible to have real-time pathogen air concentrations during a biological attack, the objective in the retrospective scenario is to use surface samples to infer what exposure and risk resulted from the release. Thus, the following discussion develops relationships between pathogen concentrations on surfaces and average dose.

After an aerosol release, the amount of pathogens in the air (M_air_), on the touched surfaces (M_ts_), and on occupants' hands (M_h_) can be acquired by solving Equation 13, which is obtained by separating out the compartments which exchange microbes from Equation 4, with the resuspension process omitted because of its minimal impact over the short time period required for the aerosol release to disperse (hours) [12]:
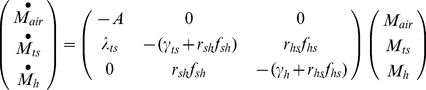
(13)whose initial conditions are:
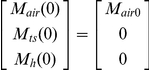



Based on the general solution listed in Equation 2, the solutions to Equation 13 are:
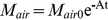
(14)

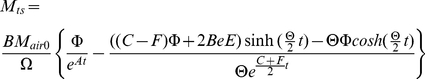
(15)

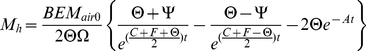
(16)where sinh and cosh are hyperbolic trigonometric functions. The coefficient A is defined by Equation 5 above while the coefficients B to F, Θ, Ω, Φ, and Ψ are defined below:
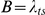


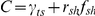


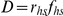


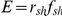


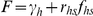


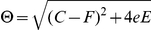


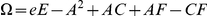


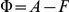


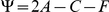



Combining Equations 14, 16 with Equation 9 (10), the total exposure dose from time t_1_ to t_2_ can be written in terms of the amount of pathogens released (Equation 17).
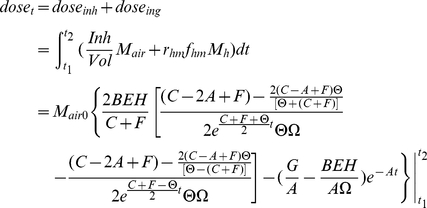
(17)where the coefficients represented by G and H are listed below:
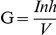


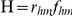



The overall risk is composed of inhalation risk and ingestion risk. In this work it is assumed that these two types of risk are independent of each other; in this case the overall risk is expressed in Equation 18:
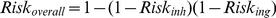
(18)In reality there is little evidence to assess the joint effects of inhalation and ingestion exposures, but this assumption is probably most defensible at low risk levels when the probability of successful colonization by both routes is low.

Equations 17 and 18 solve the forward problem of estimating risk from a known release amount. The inverse problem is to estimate the release amount from measured environmental concentrations. The amount of released pathogens (M_air0_), can be estimated by Equation 15, if the number of pathogens deposited on the touched surfaces (M_ts_) can be acquired from surface sampling and the time after release (t) is known. However, the mass on touched surface is influenced by many parameters such as touch rate and transfer rate, which are generally highly uncertain. The mass on the untracked floor is most suitable for estimating the release quantity as it provides an integration of air concentration values over time without human interference. This can be obtained by taking the expression from the fourth row of Equation 4 and substituting Equation 14 for M_air_:
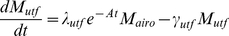
(19)and integrating it to give the release quantity, where t_m_ stands for the elapsed time when measurements are taken:
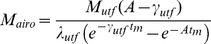
(20)


Once the release quantity (M_air0_) is known, Equations 11, 12, 17, and 18 can be used to estimate risk. Concentrations in the compartments omitted from Equation 13 (i.e., the compartments that do not transfer microbes to other compartments, namely HVAC filters, walls, nasal passages, and the external compartments) can be obtained by integrating the produce of the air concentration and the transfer rates from the air over time.

#### 2.4.2 Prospective scenario

The prospective scenario considers a case where the initial aerosol release has dissipated. However, the time scale for attenuation of microbes can be much longer on surfaces than in the air (i.e, pathogens on surfaces are not subject to attenuation by deposition or by air exchange with the exterior of the building). Thus much of the longer term risk to occupants will come from microbes on surfaces as surface can both serve as a reservoir for re-suspension into the air compartment and for exposure via fomite contact. In such cases surfaces could be sampled to assess whether a building is suitable for re-occupancy. Thus, the prospective scenario can be thought of as a re-occupancy assessment. The initial conditions are that Category A pathogens are present on the touched surfaces in a quantity equal to the area of the touched surfaces (A_surf_) multiplied by the corresponding concentration (C_surf_), which would be estimated from surface sampling (Equation 21). Thus the initial conditions can be expressed as:
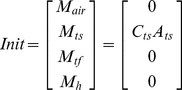
(21)


Due to the longer time scale associated with the prospective scenario, human-caused resuspension cannot be omitted. Thus, the tracked floor compartment is included in the system of equations to be solved:
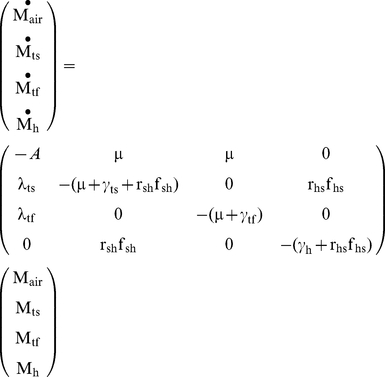
(22)


After acquiring the concentrations of the pathogen on different surfaces over time (based on Equation 2), the total exposure dose can be calculated via integration. To conservatively estimate exposure dosage, one may use the maximum exposure duration which is achieved if t_1_ = 0 and t_2_ = ∞.
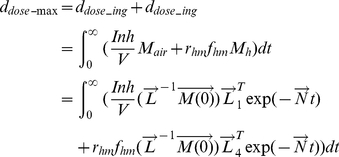
(23)


The equations derived in this section provide the exposure input for dose-response models (Equations 11 and 12) and thereby link the surface concentration of a pathogen with an occupant's future risk. This procedure is shown in Information S1, and Equations 24 and 25 (25a is for ingestion risk, 25b is for dermal contact risk; schematic figures are provided in Figures S1 and S2) are the resulting approximate solutions for the exponential dose-response model at low doses (i.e., where the Taylor series approximation is accurate. A Comparison between approximated equations and simulated results is provided in Information S1, table 1):
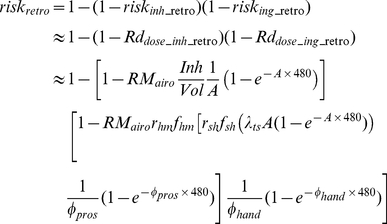
(24)

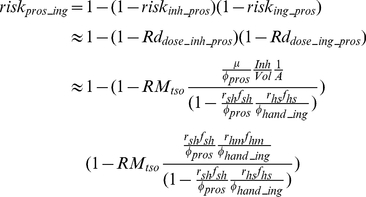
(25a)

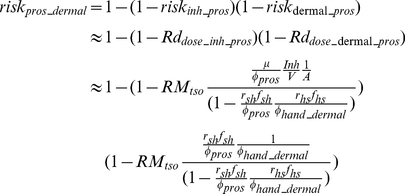
(25b)where r_hm_f_hm_ is set to 1 for dermal contact, A is defined by Equation 5, and ϕ_pros_, and ϕ_hand_ are given by:
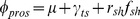
(26)

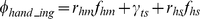
(27a)

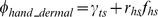
(27b)


For the beta-Poisson model these equations would hold at low dose, except that R would be replaced by α/β. At higher doses (where the Taylor series linearization does not hold) one would compute the exposure dose using Equations K and N from Information S1, and then input this dose into the appropriate dose response model.

### 2.5 Model Inputs

Environmental decay rates, best fit dose-response models, and dose response parameters for different pathogens are listed in Tables 1 and 2, while other parameters such as the dimensions of the room, the operational parameters of the HVAC system, the deposition velocities of released pathogens, etc. are included in Information S2, table 2). Since the particle size of a pathogen affects its deposition velocity, resuspension rate, filter removal, and even dose-response coefficient [32], this study considers four different aerodynamic diameters: 1 µM, 3 µM, 5 µM, and 10 µM.

**Table 2:** Best Fit Dose-Response Model.

Pathogen	Strain information	Exposed animal and route	Dose-response function type	Best-fit Virulence coefficient^a^	Uncertainty ranges of virulence coefficients (95% Confidence Interval)	Uncertainty distributions and parameter used for virulence coefficients^b^	Source
*B. anthracis* c	ATCC 6605	Female Hartley guinea pigs (250 to 300 g), intranasal	Exponential	7.15×10^−6^	(6.26×10^−6^, 7.43×10^−6^)	Normal distribution (μ = 6.93×10^−6^, σ = 3.98×10^−7^)	[59]
*Y. pestis*	CO92	C57BL/6 mice, intranasal	Exponential	1.02×10^−3^	(9.87×10^−4^, 1.05×10^−3^)	Normal distribution (μ = 1.02×10^−3^, σ = 1.91×10^−5^)	[23]
*F. tularensis*	SCHU S-4	Monkey (4000–5000 g), aerosol	Exponential	5.32×10^−2^	(5.28×10^−2^, 5.36×10^−2^)	Normal distribution (μ = 5.32×10^−2^, σ = 2.22×10^−4^)	[60] d
*Variola major*	Yamada	Swiss Webster albino mice (age from 2 hr to 6 days), intraperitoneal	Beta-Poisson	2.31×10^−6^	(8.19×10^−7^, 4.80×10^−6^)	Normal distribution (μ = 2.65×10^−6^, σ = 1.21×10^−6^)	[61] e
Lassa	NA	pigs (180 to 300 g), aerosol	Beta-Poisson	3.58×10^−2^	(4.16×10^−4^, 5.59×10^−1^)	Log-Normal distribution (μ_ln_ = −1.69, σ_ln_ = 0.80)	[57] f

a In exponential dose-response model, R is used as virulence coefficient, while in beta-Poisson dose-response model, the ratio of α/β is used as virulence coefficient.

b The distributions are fitted to bootstrap samples of dose response parameters using @RISK [62].

c The intestinal risk is replaced by cutaneous risk since the fractions of inhalational anthrax and cutaneous anthrax were the same in the 2001 anthrax letters attacks [6].

d The data for 2.1 µm particles are used.

e The data for 4.5 µm or less in diameter are used.

f The data for the age group of 5 days and above are used.

## Results

**Figure 2:**
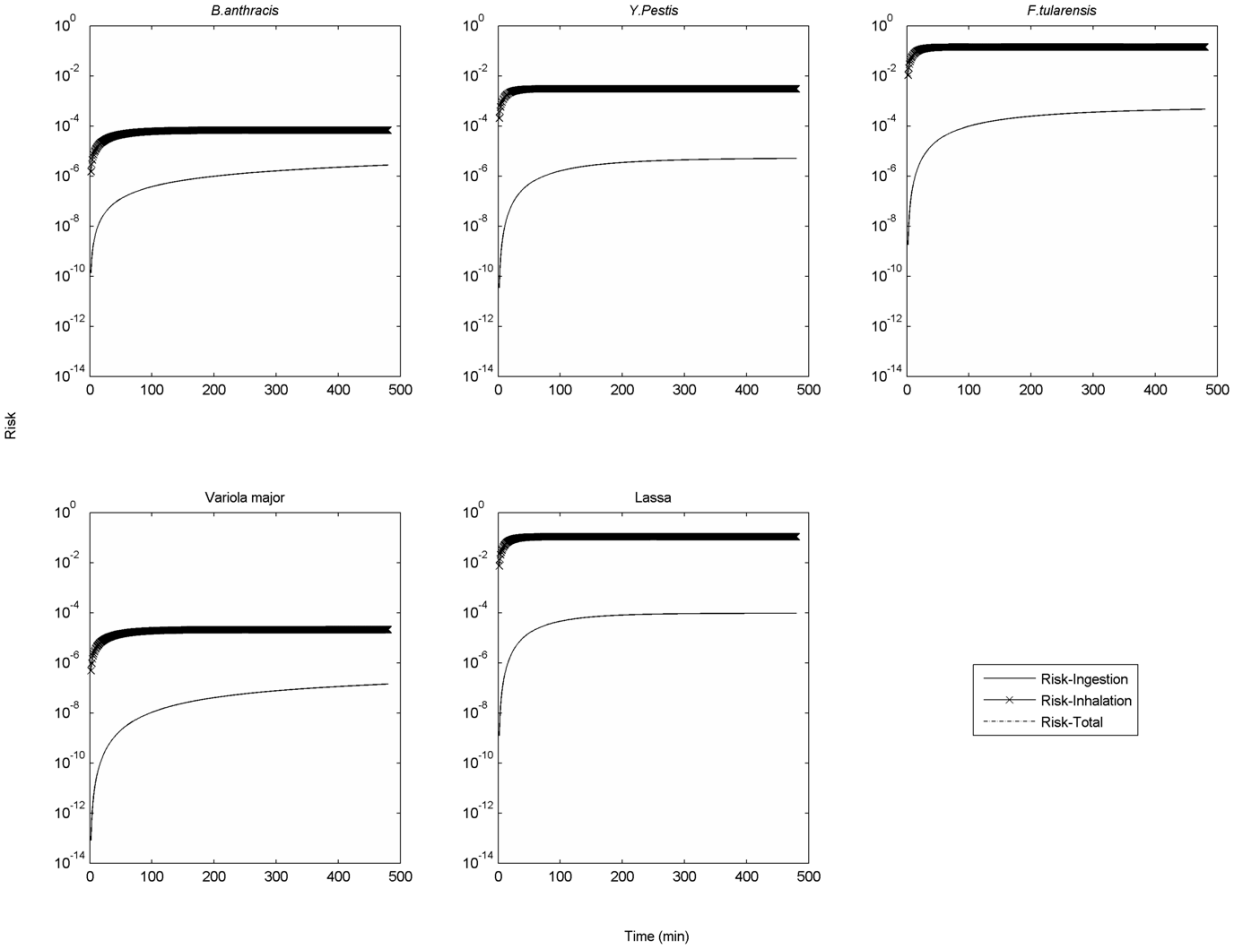
Different types of risks associated with aerosol release of 1 micron Category A pathogens. (Release quantity is 1000 unclumped pathogens. For *B. anthracis*, the ingestion risk is replaced by cutaneous risk since the fractions of inhalational anthrax and cutaneous anthrax were the same in the 2001 anthrax letters attacks [6]).

Figure 2 presents the inhalation, ingestion and overall risks associated with the aerosol release (i.e., the retrospective scenario) of 1 micron Category A pathogens. The overall risk and inhalation risk match so closely as to be indistinguishable in the graph, indicating that risk from inhalation is the main component of overall risk. Particle deposition drives the time required for this risk to reach steady state. Hence, the time to reach this asymptote is the same for different pathogens of the same size.

**Figure 3:**
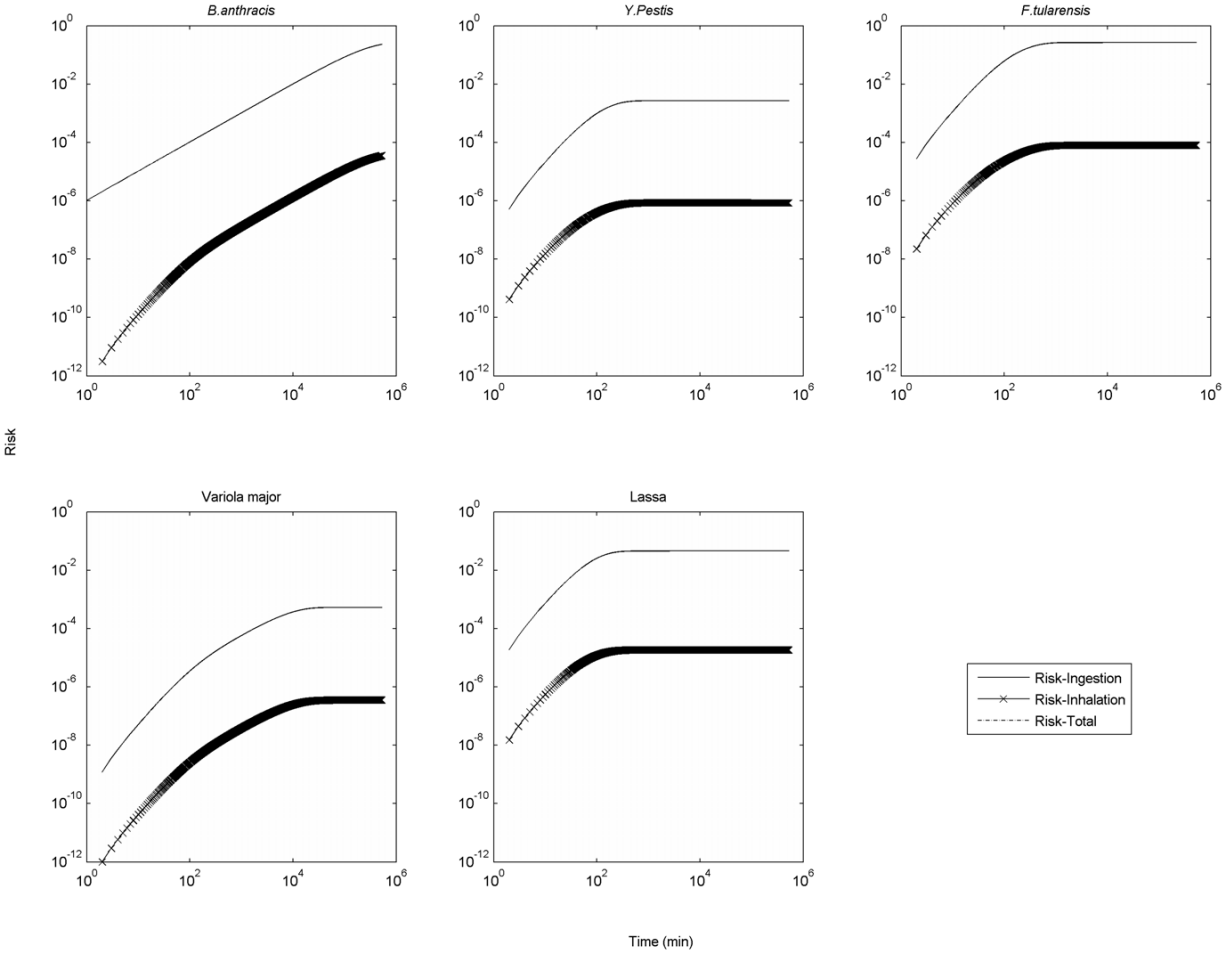
Different types of risks associated with surface release of 1 micron Category A pathogens. (Release quantity is 1000 unclumped pathogens. For *B. anthracis*, the ingestion risk is replaced by cutaneous risk since the fractions of inhalational anthrax and cutaneous anthrax were the same in the 2001 anthrax letters attacks [6]).

Figure 3 presents different types of risks associated with the presence of 1 micron Category A pathogens on surfaces (i.e., the prospective scenario). In this case total risk and ingestion risk match so closely as to be indistinguishable, indicating that risk from ingestion (dermal contact for *B. anthracis*) is the main component of overall risk. In Figure 3, the time scale over which each pathogen's overall risk reaches its asymptote varies over 4 orders of magnitude, which can be explained by the huge variability among pathogen attenuation rates.

**Figure 4:**
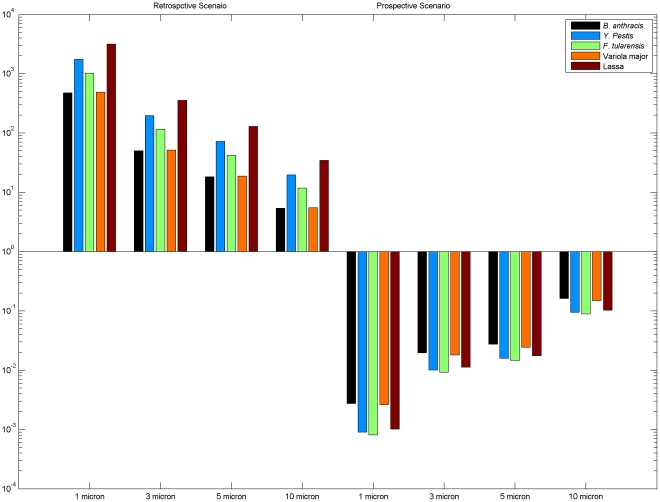
The ratio of accumulative inhalation and ingestion exposure.

To summarize which exposure routes dominate under which conditions, Figure 4 presents the ratio of accumulated inhalation and ingestion exposure. If pathogens are aerosolized (retrospective scenario), the dominant exposure route is inhalation (see also Figure 2), because inhalation is more significant for small particle sizes, which remain in the air longer before settling. If pathogens are initially present on a surface (prospective scenario), the dominant exposure route is ingestion (see also Figure 3), and this trend is most significant for small particle sizes as they are least prone to resuspension.

### 3.1 Linking pathogen concentrations to risk

**Figure 5:**
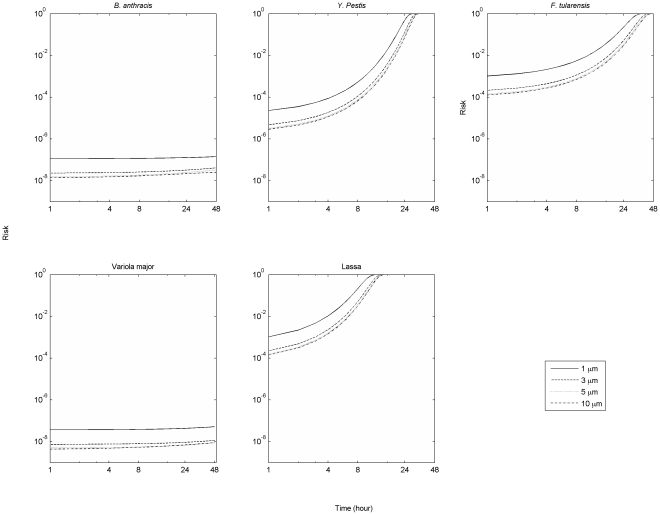
Relationship between risks to the exposed people and pathogen concentration identified from the HVAC filter. (A concentration of 10 organisms/m^2^ was found at HVAC filter at different time after an aerosol release.).

Another application of the model is to link measured pathogen concentrations on the surfaces with health risk. In the case of a persistent pathogen, a surface concentration reflects a fraction of the integral of the air concentration (provided there has been no resuspension from the surface). In contrast, for a pathogen subject to environmental decay, surface concentrations reflect both the integrated air concentrations and surface decay over time. The relationship between surface concentration and accumulated (retrospective) dose changes as pathogen concentrations attenuate on the surface over time. As deposited microbes decay, each surviving microbe becomes indicative of a larger number having been present previously. Figure 5, which depicts the retrospective risk for a concentration of 10 pathogens per m^2^ on an HVAC filter, illustrates this. If one finds the concentration of Lassa virus particles is 10 pathogens per m^2^ with a diameter of 1 µm on an HVAC system filter 1 hour after a release, this implies that occupants were subject to a risk of 1.0×10^−3^ due to the past 1 hour of exposure. The same concentration found 4 hours after the release would imply a risk close to 1.0×10^−2^, as fewer of the deposited virus remain viable after 4 hours. In reality it may not be realistic to detect pathogens in the environment on anything approaching the time scale of several hours, but this serves as an example of how great a challenge it is to use environmental samples to characterize risks associated with a pathogen that attenuates in the environment. In contrast risks associated a given concentration of *B. anthracis* (a persistent microbe) are relatively constant over time.

**Figure 6:**
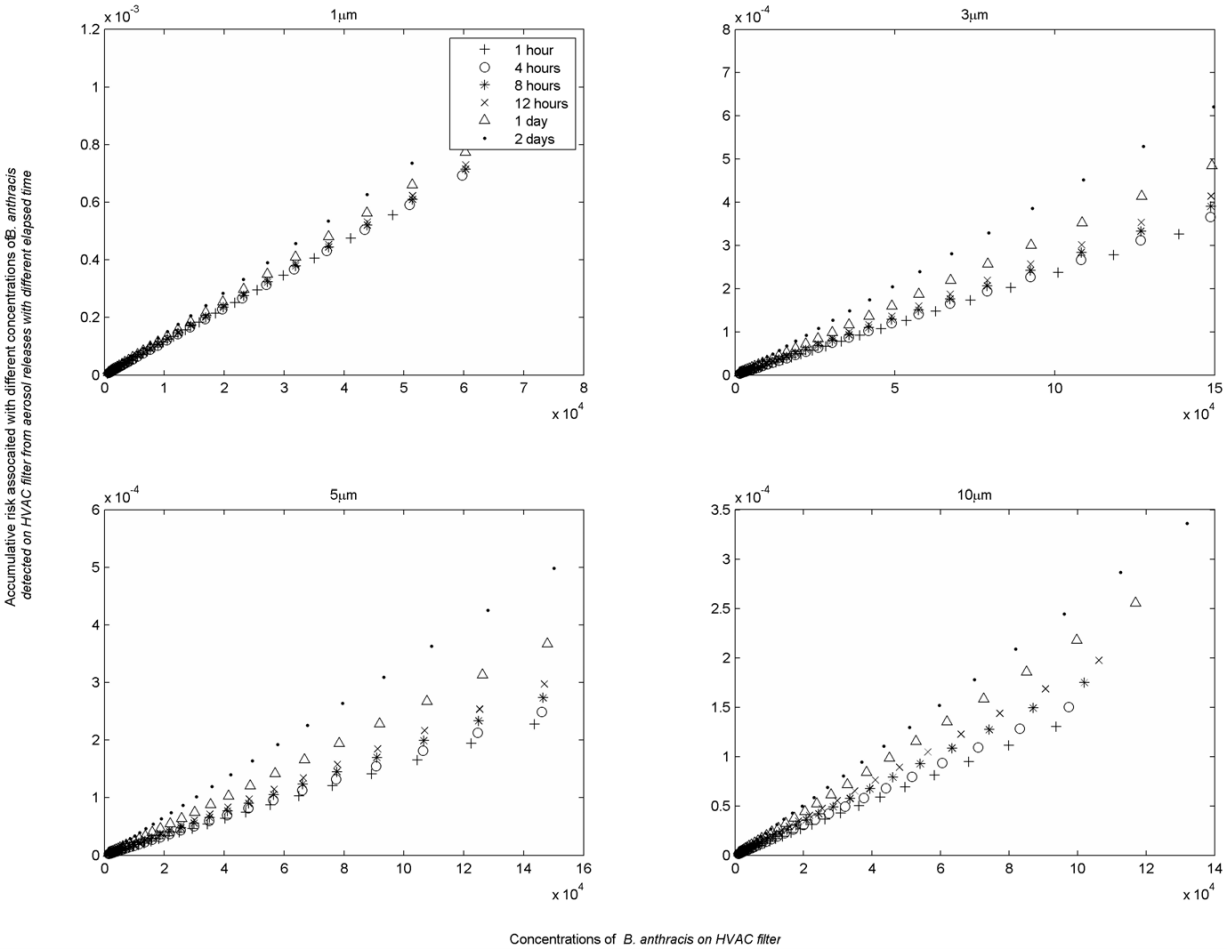
Retrospective risks associated with *B. anthracis* HVAC concentrations after an aerosol release.

**Figure 7:**
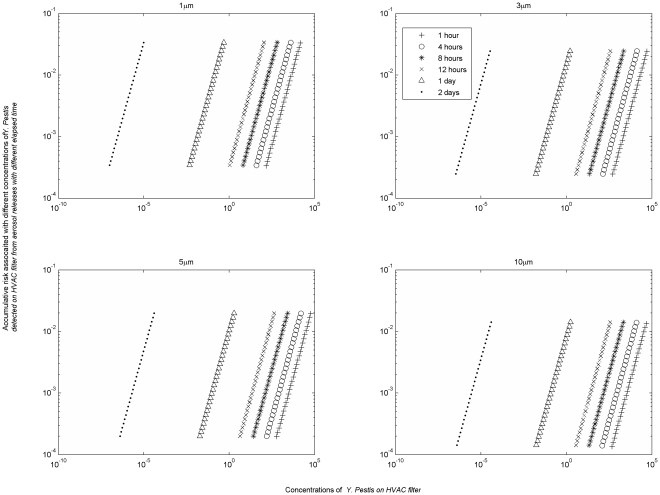
Cumulative retrospective risks associated with *Y. pestis* HVAC concentrations after an aerosol release.

The results of this modeling can be summarized in a series of charts that link surface concentration to previous exposure risk for different time periods (Figures 6 and 7 for pathogens of *B. anthracis* and *Y. Pestis*, while the rest are provided in the Information S3, Figures S3, S4, and S5). The difference between curves for the same pathogen for different time periods reflects the environmental persistence of the pathogen. A rapidly decaying pathogen will have widely separated curves to reflect that the same concentration of pathogens remaining after a longer time period implies a higher exposure risk (i.e., each pathogen remaining indicates that a greater number were present during the earlier part of the exposure period), while a persistent pathogen will have closely spaced curves as the risk to concentration relationship is relatively constant over time.

There are essentially two options for addressing prospective (re-occupancy) risk, restrict access to the contaminated site until pathogen concentrations decline to acceptable levels through natural attenuation or actively decontaminate the site. The selection of a strategy depends on the survival capability of the pathogens. If the passive decontamination approach is chosen, the time required depends on the initial concentration to reach a given residual risk target, but one can get a rough idea of the relative feasibility of this approach by comparing the time to achieve a significant concentration reduction across different pathogens. The time scale for a 6-log risk reduction due to natural attenuation for different pathogens is shown in Table 3. Values in Table 3 vary by more than 4 orders of magnitude. *B. anthracis* has a best estimate of 17,100 days or over 46 years. It is probably more appropriate to compare the upper bound as pathogens will reside in a wide variety of different microenvironments and decay rates would be expected to vary among microenvironments. Decontamination would only be achieved once even the pathogens in the more protected microenvironments have decayed. For *B. anthracis* this upper bound would be over 82 years. In contrast, a greater than 6 order of magnitude decay of Lassa would occur in less than a day. These estimates are very sensitive to the assumption of log linear decay. Deviations from log linear decay are widely reported. However in many cases a biphasic approach could be adopted in which a rapid log-linear decay rate is used for the first several days and a second, lower log-linear decay rate is used subsequently. While parameters for such biphasic attenuation models are not yet available for these pathogens, the approach presented here can be readily adapted to biphasic decay. The relevant equations would be unchanged, but the mass distribution in each compartment at the end of the first phase would constitute the initial conditions for the second phase.

**Table 3:** Time scale for a 6-log risk reduction due to natural attenuation.

Pathogen	Time (days)		
	Min	Max	Best estimate
*B. anthracis*	1.24×10^4^	3.00×10^4^	1.71×10^4^
*Y. pestis*	4.63×10^−1^	1.44×10^1^	1.27
*F. tularensis*	1.25	5.75×10^1^	2.41
*Variola major*	5.79×10^1^	1.05×10^2^	8.38×10^1^
Lassa	6.25×10^−1^	8.46×10^−1^	7.50×10^−1^

Table 4 presents the concentrations of pathogens associated with a 1 in 1000 risk. Concentrations corresponding to different risk levels can be found by multiplying these values by the desired risk level/10^−3^, provided that the risk is low enough to be approximately a linear function of exposure (which is roughly accurate for risks <10^−2^). In the retrospective scenarios (the first two columns), the concentrations become lower (standards would become more stringent) as the time after the release increases. Values for *B. anthracis* presented here are substantially lower than reported previously [12], as the previous study considered only inhalation risk, while this study considers dermal risk as well as inhalation risk for *B. anthracis*. Even if sampling could be conducted within 24 hours (which is an extremely optimistic assumption), it would be difficult to characterize risk at the 1 in 1,000 level for any of the pathogens, as this would require quantifying pathogens at levels ranging from 5–7 pathogens/m^2^ for *Variola major* to 10–11/m^2^ for Lassa.

**Table 4 pone-0032732-t004:** Concentrations of pathogens on horizontal surfaces associated with risk of 10^−3^ (Prospective exposure duration = 1 year).

Pathogen	Diameter	Concentrations (organisms/m^2^)				
		Retrospective sampling 8 hours after release	Retrospective sampling 24 hours after release	Prospective for immediate occupancy	Prospective after 24 hours access restriction	Prospective after 48 hours access restriction
*B. anthracis*	1 µM	1.63	1.73×10^−1^	2.98×10^1^	2.98×10^1^	2.98×10^1^
	3 µM	4.15	4.38×10^−1^	6.44×10^2^	6.74×10^2^	7.09×10^2^
	5 µM	7.40	7.65×10^−1^	1.88×10^3^	2.02×10^3^	2.20×10^3^
	10 µM	1.63×10^1^	1.42	7.62×10^3^	1.23×10^4^	2.11×10^4^
*Y. pestis*	1 µM	2.60×10^−3^	1.36×10^−6^	1.79×10^2^	8.99×10^6^	4.91×10^11^
	3 µM	9.80×10^−3^	2.47×10^−6^	1.78×10^2^	9.27×10^6^	5.34×10^11^
	5 µM	1.26×10^−2^	2.51×10^−6^	1.76×10^2^	9.46×10^6^	5.87×10^11^
	10 µM	1.24×10^−2^	1.72×10^−6^	1.75×10^2^	1.81×10^7^	2.03×10^12^
*F. tularensis*	1 µM	2.39×10^−4^	2.70×10^−6^	1.72	5.08×10^2^	1.60×10^5^
	3 µM	7.62×10^−4^	4.28×10^−6^	1.72	5.28×10^2^	1.73×10^5^
	5 µM	9.02×10^−4^	4.17×10^−6^	1.72	5.34×10^2^	1.82×10^5^
	10 µM	8.23×10^−4^	2.75×10^−6^	1.79	1.09×10^3^	7.03×10^5^
*Variola major*	1 µM	3.12×10^1^	7.00	1.12×10^3^	1.38×10^3^	1.62×10^3^
	3 µM	7.35×10^1^	7.69	1.35×10^3^	1.76×10^3^	2.17×10^3^
	5 µM	8.01×10^1^	7.80	1.60×10^3^	2.17×10^3^	2.74×10^3^
	10 µM	7.49×10^1^	5.84	4.77×10^3^	1.15×10^4^	2.59×10^3^
Lassa	1 µM	6.61×10^−6^	1.94×10^−11^	8.93	7.36×10^8^	7.32×10^16^
	3 µM	2.28×10^−5^	4.39×10^−11^	8.93	7.93×10^8^	8.05×10^16^
	5 µM	3.97×10^−5^	4.90×10^−11^	9.11	8.26×10^8^	8.86×10^16^
	10 µM	4.02×10^−5^	3.25×10^−11^	8.45	1.49×10^9^	3.03×10^17^

For the prospective case (columns 3–5), if a pathogen decays rapidly, most of the risk will attenuate relatively rapidly. In such cases a much less stringent concentration standard can be set if access to the building is restricted for a period after the sampling is conducted. The differences in values for different Category A pathogens are driven by virulence and environmental persistency, which are both pathogen dependent. *B. anthracis* has relatively high concentrations despite being very persistent, because it has a relatively low infectivity (proportional to parameter k from dose-response functions). The strictest concentration values are for *F. tularensis* despite its low persistence because of its high infectivity.

The concentrations associated with immediate re-occupancy are in many cases well below applicable limits of detection. For example a negative sampling result for Lassa, used to estimate the prospective risk for immediate occupancy, would not provide much confidence because the applicable standard of 9 organisms per m^2^ is well below feasible detection levels. However, a negative result coupled with a 24-hour restriction on access would provide some level of confidence as the standard for this case of 7.36×10^8^ organisms per m^2^ is readily detectable. In this latter case, demonstrating achievement of a risk target of 1 in a million (a concentration of 7.36×10^5^ organisms per m^2^ or 73.6 organisms per cm^2^) would likely be feasible as well. If one assumes that a 0.09 m^2^ surface is sampled with a recovery of 0.38 [12], [33], and a detection limit of 10 organisms, then the resulting minimum detectable pathogen concentration is 292 organisms per m^2^. Table 5 compares risks associated with this concentration across different organisms.

**Table 5 pone-0032732-t005:** Equipment detection limit associated risk*.

Pathogen	Diameter	Risk (95% confidence interval)
*B. anthracis*	1 µM	2.30×10^−2^ (5.81×10^−4^, 4.33×10^−1^)
	3 µM	6.80×10^−4^ (2.25×10^−5^, 6.54×10^−3^)
	5 µM	1.92×10^−4^ (1.00×10^−5^, 1.16×10^−3^)
	10 µM	1.17×10^−5^ (3.18×10^−6^, 2.84×10^−4^)
*Y. pestis*	1 µM	2.86×10^−3^ (9.63×10^−5^, 1.63×10^−2^)
	3 µM	2.85×10^−3^ (1.01×10^−4^, 1.58×10^−2^)
	5 µM	2.84×10^−3^ (1.04×10^−4^, 1.51×10^−2^)
	10 µM	2.69×10^−3^ (1.04×10^−4^, 1.37×10^−2^)
*F. tularensis*	1 µM	2.57×10^−1^ (1.54×10^−2^, 9.17×10^−1^)
	3 µM	2.55×10^−1^ (1.58×10^−2^, 8.96×10^−1^)
	5 µM	2.54×10^−1^ (1.60×10^−2^, 8.65×10^−1^)
	10 µM	2.31×10^−1^ (1.56×10^−2^, 8.06×10^−1^)
*Variola major*	1 µM	2.79×10^−4^ (9.72×10^−6^, 6.10×10^−4^)
	3 µM	2.43×10^−4^ (7.84×10^−6^, 5.06×10^−4^)
	5 µM	2.14×10^−4^ (5.93×10^−6^, 4.06×10^−4^)
	10 µM	7.35×10^−5^ (3.42×10^−6^, 3.32×10^−4^)
Lassa	1 µM	5.43×10^−2^ (1.04×10^−2^, 7.00×10^−1^)
	3 µM	5.43×10^−2^ (1.09×10^−2^, 7.00×10^−1^)
	5 µM	5.42×10^−2^ (1.12×10^−2^, 6.99×10^−1^)
	10 µM	5.26×10^−2^ (1.12×10^−2^, 6.95×10^−1^)

* It is assumed that the detection limit is 10 organisms which comes from sampling a 0.09 m^2^ surface with the pathogen concentration 292 organisms per m^2^ and the recovery rate is 0.38 [12].

### 3.2 Parameter uncertainties

**Figure 8:**
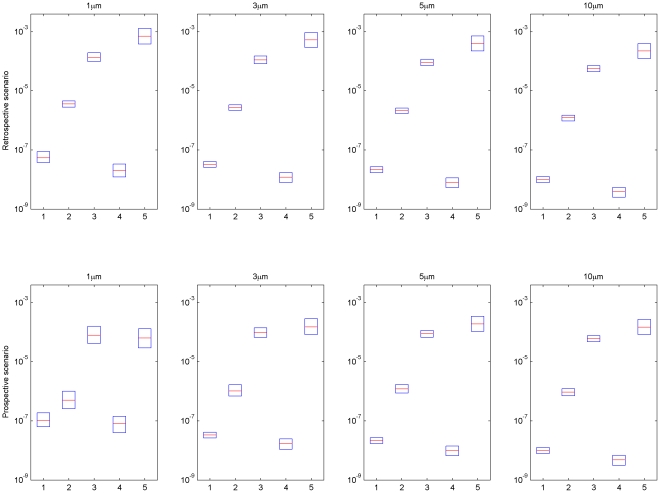
Risk and uncertainty for different pathogens associated with an aerosol release over 8 hours (retrospective scenario) and with a surface release over an infinite time (prospective scenario). Medians shown in red, 1^st^ and 3^rd^ quartiles in blue. For input uncertainty distributions see Tables 1–2 of the main text and Information S2, table 2. (1. *B. anthracis*, 2. *Y. pestis*, 3. *F. tularensis*, 4. *Variola major*, and 5. Lassa).

Another objective of this study is to compare risk and uncertainties across different pathogens. The development of explicit formulae for exposure, that is Equations 24 and 25a(b), greatly simplifies uncertainty analysis. Using the input distributions listed in Table 1 and Table 2 with Equations 24 and 25a(b), a Monte Carlo analysis was carried out using to propagate uncertainties in input parameters through to uncertainties in risks for different pathogens. Results from retrospective and prospective scenarios are presented in the form of box plots (Figure 8). The relative risk presented by different pathogens in an air release are largely determined by their dose-response parameters, because the exposure duration in air is limited by the particle deposition rate (which is the same across different pathogens) rather than the decay rate. However, air decay rate does have an impact, when decay is rapid enough to occur over the time scale during which particulates are typically suspended (minutes to hours depending on the diameter of the particles) which is the case for *Y. pestis*, *F. tularensis*, and Lassa.,The relative risks for different pathogens in a surface release are affected by both fomite decay rates and dose-response parameters. In general the risk from releasing the same amount of pathogens can be ranked as Lassa, *F. tularensis*, *Y. Pestis*, *B. anthracis*, and *Variola major* (in decreasing order). This analysis does not include secondary transmission risks (which may be particularly important for all but *B. anthracis* and *F. tularensis* [34]) and as such does not capture a critical component of risk for pathogens, such as *Variola major*, which are subject to secondary transmission. Instead it addresses the question as to which pathogens are subject to the greatest uncertainty in setting surface concentration standards for primary exposure. Uncertainties presented by Lassa are highest across most of the cases (extending over roughly an order of magnitude), indicating that this organism may be a priority for further study (pending consideration of factors such as its likely use in an attack).

Correlations between the input parameter values and the model output (risk) are used to assess the importance of uncertainties in different parameters. These correlations were computed separately for each pathogen, for ingestion and inhalation risk for both the retrospective and prospective scenarios for all four particle sizes considered. Table 6 summarizes the 3 most important uncertain inputs by exposure pathway and scenario for each pathogen (with the range of values across the four particle sizes shown in brackets). Detailed results are included in the Information S4, tables 3–7). Uncertainties in mass transfer fraction from surface to hand (f_sh_) have major impacts on ingestion dose, and uncertainty in the breathing rate (Inh) plays an important role in determining the inhalation dose in the retrospective scenario. In the prospective scenario, the ingestion dose is most closely related to the mass transfer fraction from surface to hand (f_sh_), while the inhalation dose is most closely related to the pathogen resuspension rate (μ_2_).

**Table 6 pone-0032732-t006:** Parameter uncertainties with most influence on risk.

Pathogen	Retrospective scenario		Prospective scenario	
	Ingestion risk	Inhalation risk	Ingestion risk	Inhalation risk
*B. anthracis*	Dose-response coefficient (r) (0.66–0.76)	Air change rate (ACH) (0.31–0.72)	Dose-response coefficient (r) (0.63–0.87)	Air change rate (ACH) (0.44–0.75)
	Mass transfer fraction from surface to hand (f_sh_) (0.21–0.36)	Breathing rate (Inh) (0.27–0.65)	Mass transfer fraction from surface to hand (f_sh_) (0.16–0.32)	Breathing rate (Inh) (0.14–0.53)
	Air change rate (ACH) (0.081–0.21)	Density of the particle (ρ_p_) (0.052–0.61)	Resuspension rate (μ_2_) (0.047, 0.29)	Resuspension rate (μ_2_) (0.022–0.32)
*Y. pestis*	Decay rate on fomite (γ_f_) (0.56–0.61)	Breathing rate (Inh) (0.73–0.78)	Decay rate on fomite (γ_f_) (0.53–0.56)	Decay rate on fomite (γ_f_) (0.51–0.63)
	Mass transfer fraction from surface to hand (f_sh_) (0.47–0.51)	Air change rate (ACH) (0.28–0.52)	Mass transfer fraction from surface to hand (f_sh_) (0.34–0.38)	Resuspension rate (μ_2_) (0.21–0.35)
	Density of the particle (ρ_p_) (0.12–0.24)	Density of the particle (ρ_p_) (0.026–0.54)	Hand-surface contacting rate (r_hs_) (0.071–0.079)	Breathing rate (Inh) (0.15–0.18)
*F. tularensis*	Mass transfer fraction from surface to hand (f_sh_) (0.44–0.67)	Decay rate in the air (γ_air_) (0.46–0.75)	Decay rate on fomite (γ_f_) (0.64–0.65)	Decay rate on fomite (γ_f_) (0.42–0.59)
	Decay rate in the air (γ_air_) (0.23–0.43)	Breathing rate (Inh) (0.35–0.69)	Mass transfer fraction from surface to hand (f_sh_) (0.41–0.47)	Resuspension rate (μ_2_) (0.18–0.33)
	Decay rate on fomite (γ_f_) (0.33–0.49)	Decay rate on fomite (γ_f_) (0.18–0.42)	Hand-surface contacting rate (r_hs_) (0.12–0.13)	Decay rate in the air (γ_air_) (0.14–0.26)
*Variola major*	Mass transfer fraction from surface to hand (f_sh_) (0.45–0.67)	Dose-response coefficient (r) (0.44–0.73)	Mass transfer fraction from surface to hand (f_sh_) (0.60–0.67)	Dose-response coefficient (r) (0.30–0.61)
	Dose-response coefficient (r) (0.38–0.54)	Air change rate (ACH) (0.19–0.54)	Dose-response coefficient (r) (0.51–0.57)	Air change rate (ACH) (0.25–0.45)
	Air change rate (ACH) (0.11–0.37)	Breathing rate (Inh) (0.25–0.40)	Resuspension rate (μ_2_) (0.15–0.46)	Resuspension rate (μ_2_) (0.25–0.39)
Lassa	Dose-response coefficient (r) (0.60–0.70)	Dose-response coefficient (r) (0.69–0.87)	Dose-response coefficient (r) (0.72)	Dose-response coefficient (r) (0.61–0.80)
	Mass transfer fraction from surface to hand (f_sh_) (0.40–0.47)	Breathing rate (Inh) (0.26–0.28)	Mass transfer fraction from surface to hand (f_sh_) (0.51)	Breathing rate (Inh) (0.17–0.23)
	Decay rate in the air (γ_air_) (0.057–0.21)	Decay rate in the air (γ_air_) (0.082–0.31)	Resuspension rate (μ_2_) (0.26–0.40)	Decay rate in the air (γ_air_) (0.089–0.24)

Correlation coefficients between selected parameters and risks. Correlations were computed separately for each of four modeled particle sizes (1, 3, 5, and 10 µM diameter particles), and the smallest and the largest coefficients across the four modeled particle sizes are listed in the brackets. (Raw data are included in Information S4).

**Table 7:** Properties of parameters uncertainty.

Authors' priority	Parameter	Symbol	Uncertainty vs. Variability	Generality	Researchable	Percentage in the top 3 uncertainty parameters among retrospective scenario (%)+	Percentage in the top 3 uncertainty parameters among prospective scenario (%)+
High	Mass transfer fraction from surface to hand	f_sh_	Both	Similarities expected	Yes	0	33
High	Dose-response coefficients	k	Both	Pathogen specific	Difficult	13	20
High	Resuspension rate	μ_2_	Both	Similarities expected	Yes	0	20
High	Hand-surface contacting rate	r_hs_	Both	Similarities expected	Yes	0	13
Moderate	Decay rate on fomite	γ_f_	Both	Pathogen specific	Yes	7	13
Moderate	Decay rate in the air	γ_air_	Both	Pathogen specific	Yes	13	0
Low	Breathing rate	Inh	Variability	Common across pathogen	Yes	33	0
Low	Air change rate	ACH	Variability	Common across pathogen	Yes	20	0
Low	Density of the particle	ρ_p_	Variability	Common across pathogen	Yes*	13	0

* Density can readily be measured but it is not clear that laboratory values could reflect density in an actual release.

+ The percentages in the retrospective scenario are based on inhalation risk in the retrospective scenario, while the percentages in the prospective scenario are based on ingestion risk in the prospective scenario of Table 6.

As noted above this analysis captures only one aspect of uncertainty, that of uncertainty in primary exposure. This may be the appropriate framework for pathogens that are not subject to secondary transmission as well as for decisions where one seeks to cut off environmental transmission of a pathogen after a widespread environmental contamination event. Additional risk and uncertainty would be applicable for decisions where secondary transmission is a concern.

## Discussion

This study presents an integrated fate and transport, dose-response model to estimate the inhalation and ingestion risks associated with environmental pathogens. Scenarios to estimate the past risk and to predict future risk are introduced. A reduced form model is developed and used to compare risks and uncertainties for different pathogens. Efforts to develop an internet-based platform for the dissemination of microbial risk assessment tools such as this are in progress (http://wiki.camra.msu.edu/index.php?title=Main_Page).

In addition, this study also identified important parameter uncertainties in risk assessment models. Specifically, the input-output correlations presented in Table 6 indicate which parameter uncertainties have the greatest effect on risk estimates. However, several other factors must be considered in settling research priorities. Whether the high correlation is due to variability or epistemic uncertainty is one such factor. Parameters such as inhalation rate and air exchange rate will vary considerably from person to person and from building to building, respectively. However, they are not subject to great epistemic uncertainty. The ranges within these parameters vary have already been well characterized. Additional research would not reduce the inherent variability in such parameters but only serve to further characterize an already well-characterized variability distribution.

Another factor to consider is whether a particular parameter is common across pathogens such that a study of a single surrogate organism might be helpful in improving risk assessments for multiple pathogens. Strictly speaking, any parameter can be considered pathogen specific. However, some distinctions can perhaps be made between dose-response parameters and environmental decay rates, both of which are observed to vary over orders of magnitude and depend on very complex pathogen-host and pathogen-environment interactions, and general physical transfer rates, such as surface-hand and hand-surface transfer fractions and re-aerosolization rates, which might vary less from pathogen to pathogen.

A third consideration is the extent to which an uncertainty is reducible by further research. Dose-response is an example of an uncertainty that is difficult to reduce through research. In part this is due to cost, as such research generally requires vertebrate animals and extensive biosafety precautions. There are other more fundamental challenges as well. Laboratory experiments 1) must be conducted at high doses with limited numbers of animals, leaving great uncertainty as to the effects of lower doses; 2) generally do not consider the effects of previous exposures, which might greatly affect the dose response coefficients; and 3) must be conducted with animal models that may not accurately represent human dose response. Despite these limitations, further animal studies would at least reduce the confidence intervals for the dose-response parameters used here. These dose-response model parameter uncertainties are the uncertainties reflected in the correlations summarized in Table 6 (i.e., applicability of the animal model to humans and validity of extrapolation from high to low dose were not addressed by this analysis), which means that further animal dosing studies would effectively reduce the uncertainty considered here. Thus, dose-response uncertainty is considered by the authors to be researchable, although the difficulties and expense of working with vertebrate animals with extensive biosafety precautious are significant.

As an example of how one might integrate these different factors, Table 7 summarizes the authors' view of future research priorities based on these different factors. In Table 7, The percentages in the right hand columns indicate the frequency with which the parameter was one of the top three sources of uncertainty for different pathogens (the retrospective scenario percentages are based on inhalation risk, and the prospective scenario percentages are based on ingestion risk). A low research priority for research is assigned to all three parameters subject to variability rather than epistemic uncertainty: breathing rate, density, and air exchange rate. The remaining 6 parameters all were judged to be subject to epistemic uncertainty. The degree of “Generality” (divided into 3 categories in order of priority: common across pathogens, similarities expected, pathogen specific) was an important factor in distinguishing among high and medium priority parameters, with both of the medium priority parameters (decay on fomites and decay in the air) considered to be pathogen specific. Three of the high priority parameters (resuspension rate, hand surface contact rate, and mass transfer fraction for surface to hand) were ranked highly partly because similarities across organisms would be expected making surrogate research more generally relevant and partly because the input-output correlations indicated they were important parameters. Dose-response parameters were given high priority for research despite being judged both pathogen-specific and difficult to research, because these parameters were relatively frequently among the parameters responsible for the greatest uncertainty in risk (13% of retrospective cases and 20% of prospective cases).

Judgments listed in Table 7 are all based on the authors' understanding and previous experience. The intent is to provide an example framework for integrating the computational results provided by the model with broader considerations that influence the costs and benefits expected from future research. The sources of input into this ranking process should be broadened by scientifically collecting opinions from experts in the future [35].

The fate and transport model is based on the assumption that pathogens are instantly uniformly mixed in a compartment. This fails to capture the short-term dynamics associated with the immediate vicinity of a release. For example, surface samples might not be reflective of the localized high concentrations associated with opening a letter containing pathogens and might underestimate risk in this case. A more detailed approach, such as computational fluid dynamics, would be a useful extension to this study.

The study also considers risk from a release of only one pathogen. Little information is available on the effects of mixtures of pathogens. This approach would be most valid at low risk levels when interactions among pathogens, such as successful colonization by more than one pathogen would be unlikely.

Another assumption is that pathogen attenuation rate outside the host is log linear over time. In reality microorganisms often exhibit “tailing” in which a small, highly resistant subpopulation attenuates at a very low rate. Thus the assumption of log-linear decay may not be health protective. Accordingly the values calculated here are not intended as suggested environmental standards. These calculations are provided to illustrate the suggested approach and to allow a comparison of uncertainties so that future research can be prioritized.

This study modeled environmental fate and transport using a small number of homogeneous compartments when in reality surfaces may vary in characteristics, such as the frequency with which they are touched, the rate at which pathogens attenuate (influenced in turn by relative humidity, intensity of ultraviolet light, etc. [10]), and the ease with which pathogens are re-aerosolized or transferred to hands from them. Modeling these heterogeneities may improve our understanding of pathogen fate and transport in the environment but would require detailed parameter inputs beyond what are currently available in the literature. Such heterogeneities might provide protected microenvironments that could allow pathogens to persist longer and present greater health risks than estimated here, which makes this a priority for future research.

The framework developed here may help inform whether active decontamination is required after a release. If a pathogen with a slow environmental attenuation rate is released (i.e., *B. anthracis*), then environmental decontamination may be required. In contrast, if a pathogen with fast environmental attenuation rate is released (eg., Lassa), the decision maker may opt to restrict access to the contaminated site until the residual risk declines to a level judged acceptable for re-occupancy. The choice between active decontamination and passive attenuation involves comparing the costs of remediation and opportunity costs of restricting access to the building. While previous research has addressed policy options for bioterrorism, this research has not considered the opportunity costs of removing buildings from service [36], [37]. Thus, further study is needed to inform the choice between active remediation and passive attenuation.

This analysis considered viable organisms. However, the environmental concentrations which would be used as inputs to the risk models developed here would likely be measured by quantitative PCR (qPCR), which has been proven effective in quantifying biological warfare agents (i.e., *B. antracis*, and *Y. pestis*) due to its rapid, early, and accurate results [38]. Despite the advantages of qPCR analysis, several knowledge gaps need to be addressed The first is that the qPCR does not distinguish between living or dead pathogens. While researchers have identified assays to discriminate between viable and dead fecal *bacteroidales* bacteria, similar methods have not been applied to Category A pathogens [39], [40]. Second there is little information on the decay of the qPCR signal over time, which would be an essential parameter for the retrospective assessment of risk after a release. Thus, studies are needed to quantify parameters such as, the efficiency of DNA extraction, the degradation of nucleic acids overtime, and the reactivity of primer and probe [33], [41], [42].

## Supporting Information

Figure S1 **Pathogen flow for estimating the inhalation dose in the prospective scenario.**
(TIF)

Figure S2 **Pathogen flow for estimating the ingestion dose in the prospective scenario.**
(TIF)

Figure S3 **Cumulative retrospective risks associated with F. tularensis HVAC concentrations after an aerosol release.**
(TIF)

Figure S4 **Cumulative retrospective risks associated with Variola major HVAC concentrations after an aerosol release.**
(TIF)

Figure S5 **Cumulative retrospective risks associated with Lassa HVAC concentrations after an aerosol release.**
(TIF)

Information S1 **The simplification of risk assessment model.**
(DOC)

Information S2 **Model Inputs.**
(DOC)

Information S3 **Cumulative retrospective risks associated with pathogens (** ***F. tularensis*** **,** ***Variola major*** **and Lassa) HVAC concentrations after an aerosol release.**
(DOC)

Information S4 **Correlation coefficients between input parameters and different pathogens.**
(DOC)
