# Correction: Prioritizing Risks and Uncertainties from Intentional Release of Selected Category A Pathogens

**DOI:** 10.1371/annotation/4565764c-51fd-42c3-b1b2-f74b90c08666

**Published:** 2012-09-27

**Authors:** Tao Hong, Patrick L. Gurian, Yin Huang, Charles N. Haas

There were errors in the published affiliations. The correct affiliations are:

Tao Hong^1^*, Patrick L. Gurian^1^, Yin Huang^1^, Charles N. Haas^1^

1. Department of Civil, Architectural, and Environmental Engineering, Drexel University, Philadelphia, Pennsylvania, United States of America

