# Supplementary figures and images for: Prioritizing Risks and Uncertainties from Intentional Release of Selected Category A Pathogens
Source: PLoS One. 2012 Mar 6;7(3):e32732. doi: 10.1371/journal.pone.0032732 (PMC3295774; doi:10.1371/journal.pone.0032732)

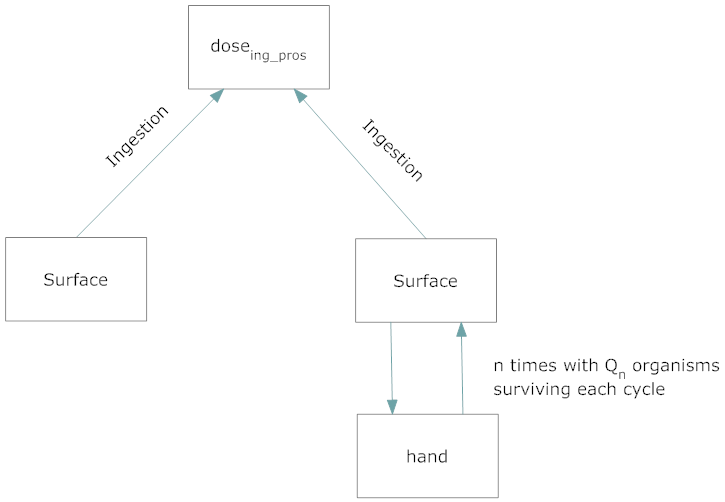

Supplement: Figure S1 — Pathogen flow for estimating the inhalation dose in the prospective scenario. (TIF) [file pone.0032732.s001.tif]

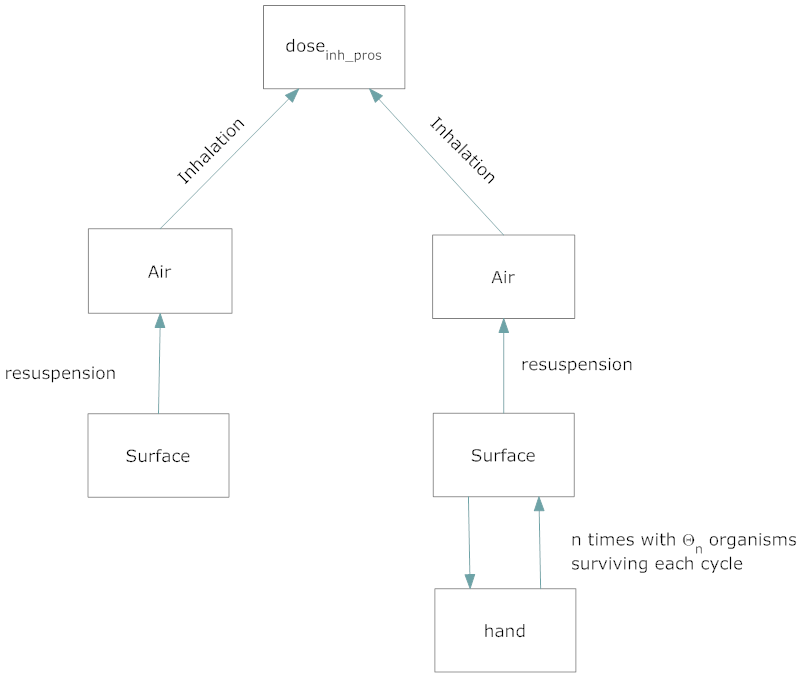

Supplement: Figure S2 — Pathogen flow for estimating the ingestion dose in the prospective scenario. (TIF) [file pone.0032732.s002.tif]

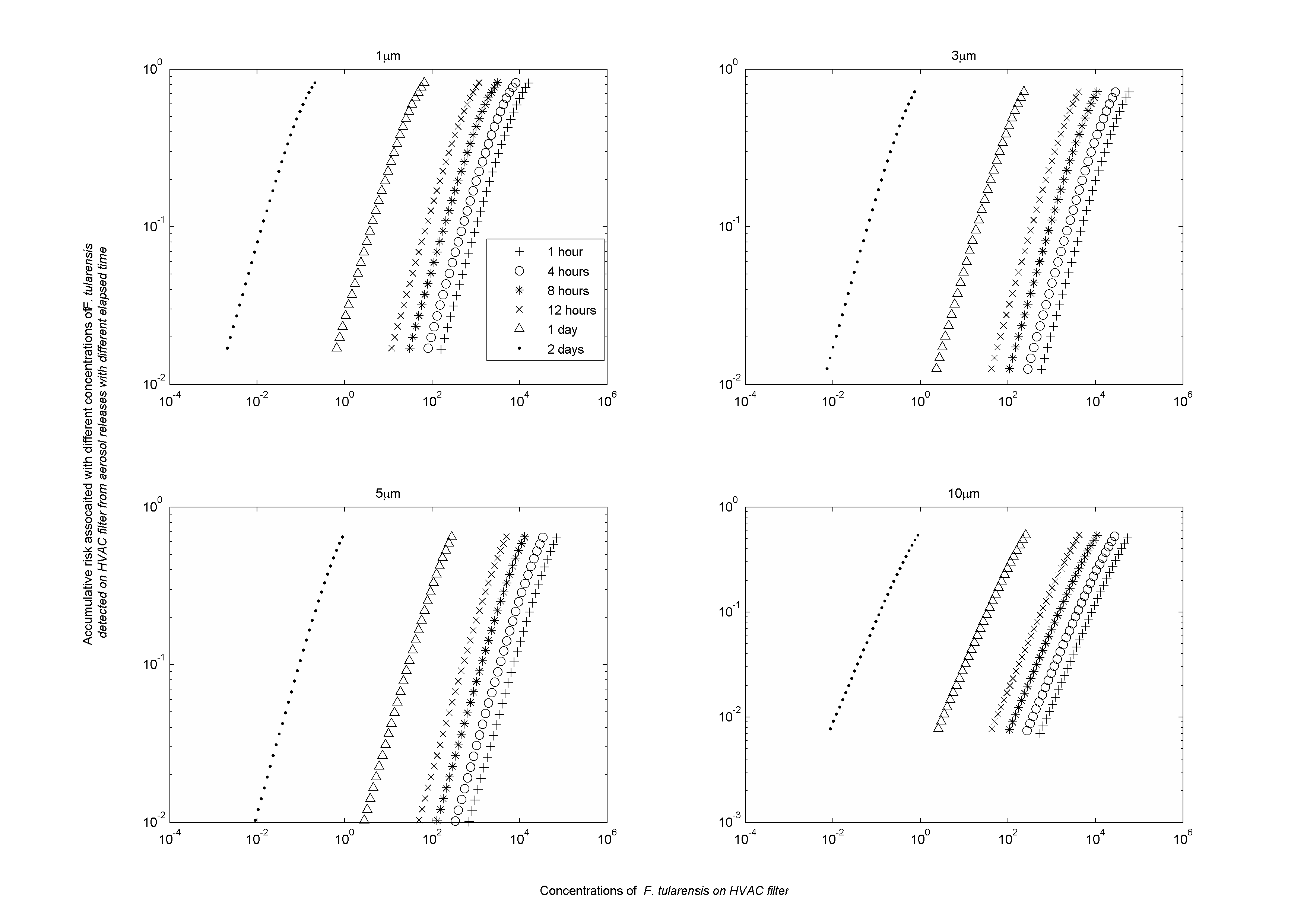

Supplement: Figure S3 — Cumulative retrospective risks associated with F. tularensis HVAC concentrations after an aerosol release. (TIF) [file pone.0032732.s003.tif]

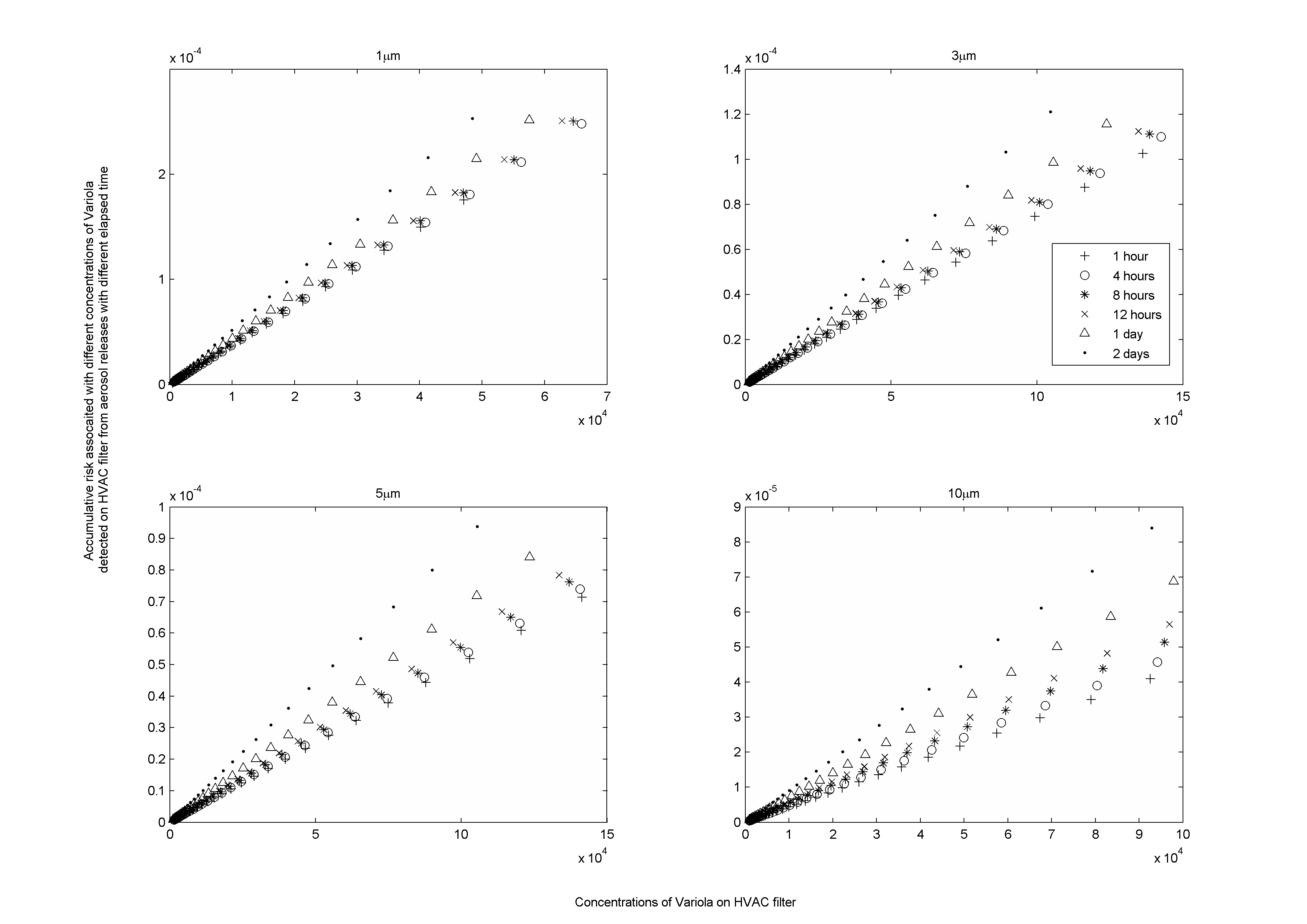

Supplement: Figure S4 — Cumulative retrospective risks associated with Variola major HVAC concentrations after an aerosol release. (TIF) [file pone.0032732.s004.tif]

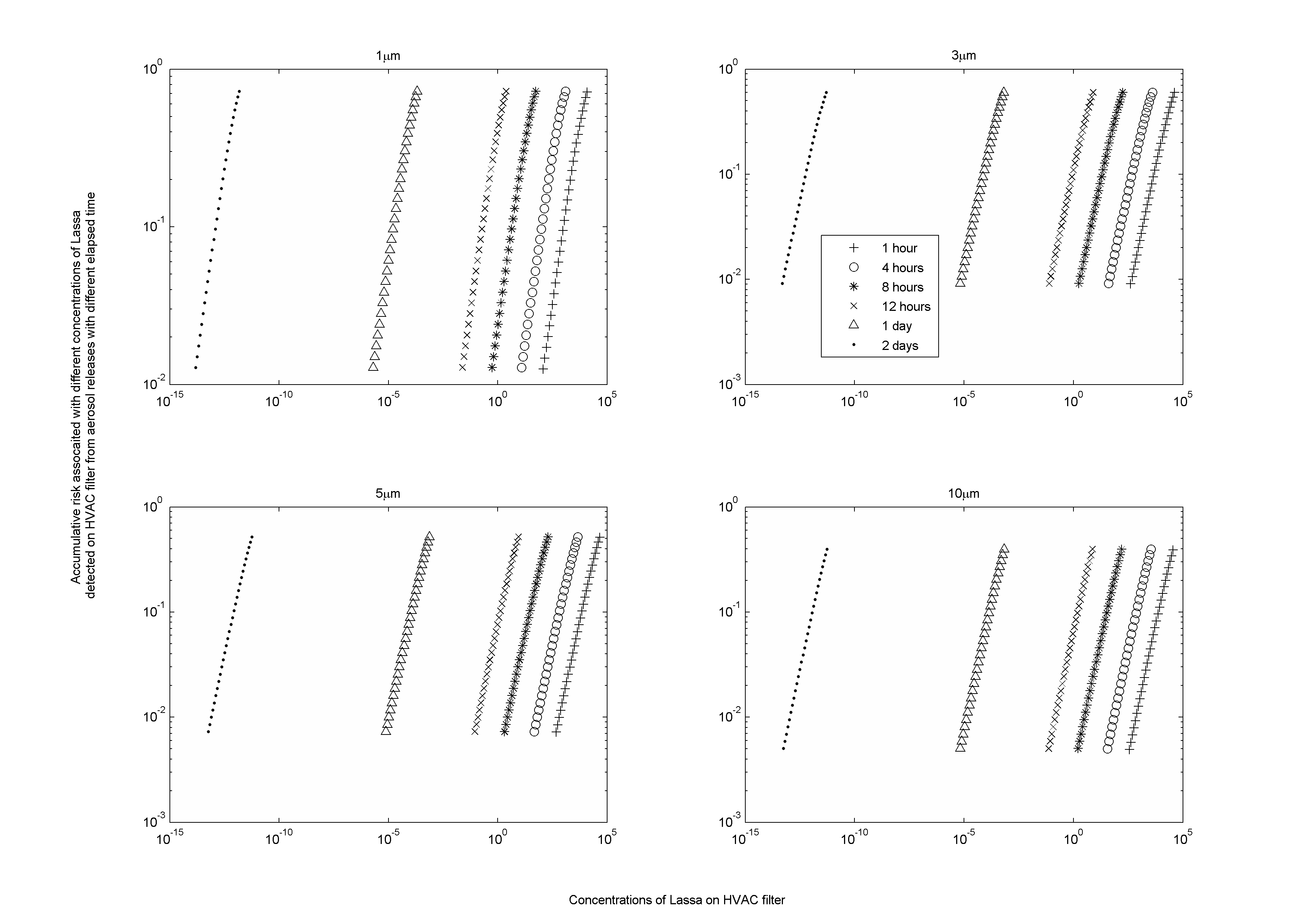

Supplement: Figure S5 — Cumulative retrospective risks associated with Lassa HVAC concentrations after an aerosol release. (TIF) [file pone.0032732.s005.tif]
