# Supplementary material for: Prioritizing Risks and Uncertainties from Intentional Release of Selected Category A Pathogens
Source: PLoS One. 2012 Mar 6;7(3):e32732. doi: 10.1371/journal.pone.0032732 (PMC3295774; doi:10.1371/journal.pone.0032732)
Supplement: Information S1 — The simplification of risk assessment model. (DOC) [file pone.0032732.s006.doc]

Prioritizing Risks and Uncertainties from Intentional Release of Selected Category A Pathogens

Tao Hong*^1^, Patrick L. Gurian^2^, Yin Huang^3^, and Charles N. Haas^2^

1. National Exposure Research Laboratory, EPA, Athens, GA, USA, 2. Department of Civil, Architectural, and Environmental Engineering, Drexel University, Philadelphia, PA, USA, 3. Office of Biostatistics and Epidemiology, FDA, Rockville, MD, USA

*hongtao510@gmail.com

**SUPPORTING INFORMATION**

**Supporting Information S1** The simplification of risk assessment model

**Supporting Information S1**

This supporting information describes how the system of equations given by Equation 1 in the main body of the paper can be simplified to allow reduced form solutions to be developed.

**Retrospective scenario**

In the retrospective scenario, resuspension makes a negligible contribution to dose, due to the relatively short exposure period compared the rates of resuspension (in this case the exposure period is assumed to be is 8 hours, the duration of a working day). Neglecting re-suspension separates the air compartment from the effects of other compartments so that air concentration follows a simple first order decay model. The inhaled dose can be calculated as:

(Eq. A)

where

(Eq. 31)

The ingested pathogens (doseing_retro) are the organisms which deposit on the touched surface (M_ts_) from the initial release in the air (M_air0_) (Equation B):

(Eq. B)

remain alive until being transferred to the hand (M_hand_) (Equation C):

(Eq. C)

and are ingested (doseing_retro) during surface-hand-mouth contact in the exposure period (Equation D):

(Eq. D)

where

(Eq. 32)

(Eq. 33)

To simplify the calculation, three assumptions are made during the derivation of Equation D: 1) pathogen resuspension and back transfer from hands to the surface are omitted due to their relatively low rates resulting in small fractions being back transferred, which is also health conservative; 2) all integration steps are from t=0 to t=a which provides an upper bound on the amount of pathogen transferred to the next step; 3) the pathogens will not be transferred to hands until depositing on the touched surface, and the pathogens will not be ingested until they are transferred to hands.

**Prospective scenario**

In the prospective scenario, the majority of the inhaled dose (doseinh_pros) comes from two sources (Figure S1). The first source consists of organisms that are inhaled right after being resuspended (doseinh1_pros) (Equation E).

(Eq. E)

The second are those organisms which experienced a certain number of "surface-hand-surface" travels before being resuspended and inhaled doseinh2_pros (Equation F).

(Eq. F)

where Θ is the total fraction of resuspended pathogens surviving a number of n "surface-hand-surface" cycles:

(G)

Θ is composed as a summation of a geometric series with element of Θ_n_, where n indexes the number of "surface-hand-surface" cycle. In the n^th^ cycle, pathogens survived from the n-1^th^ cycle (Θ_n-1_M_tso_) are first transferred to hands (Equation H), and then back transferred to the surface (Equation I).

(H)

(I)

Combine Equation H and I:

(J)

For both sources, pathogen resuspension happens before inhalation. The maximum inhalation dose is reached when the exposure duration goes to infinity (Equation K):

(Eq. K)

Similarly, the prospective ingestion dose (doseing_pros) comes from two sources (Figure S2). The first source is direct ingestion of the pathogens released on the touched surface (doseing1_pros) (Equation L):

(Eq. L)

where is the mass transferred to victims hand.

The second source is those organisms which experience a certain number of "surface-hand-surface" travels before being ingested (doseing2_pros) (Equation M), which contains the common factor Θ as described above. The maximum ingestion dose is reached when exposure goes to infinity (Equation N).

(Eq. M)

(Eq. N)

Table S1 compares the exposure dose approximated by the above-mentioned equations with the exact results from solving Equation 18. The overall risk is acquired by inputting inhalation and ingestion doses into Equation 14 separately for the retrospective (Equation 26) and the prospective scenario (Equation 27).

(Eq. 26)

(Eq. 27)


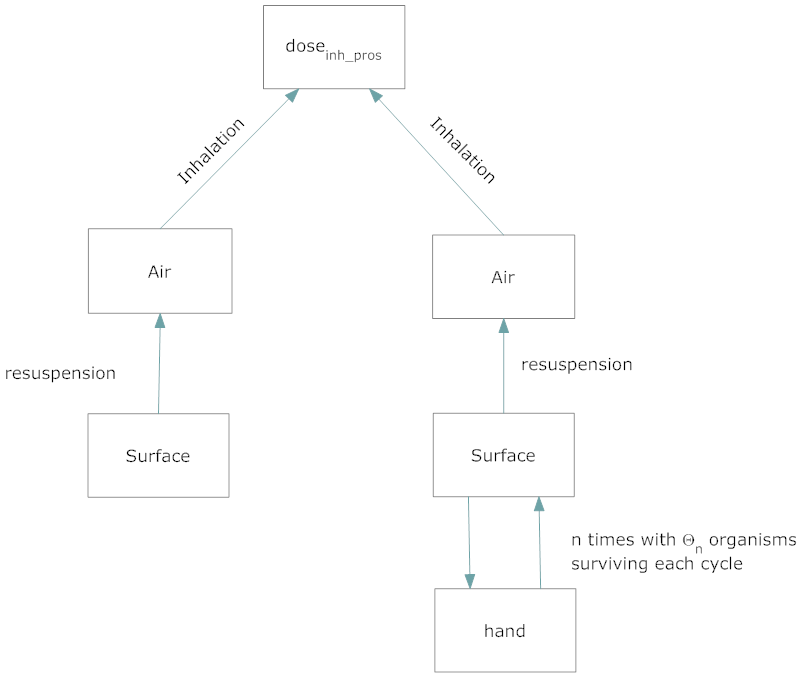


**Figure S1. Pathogen flow for estimating the inhalation dose in the prospective scenario.**


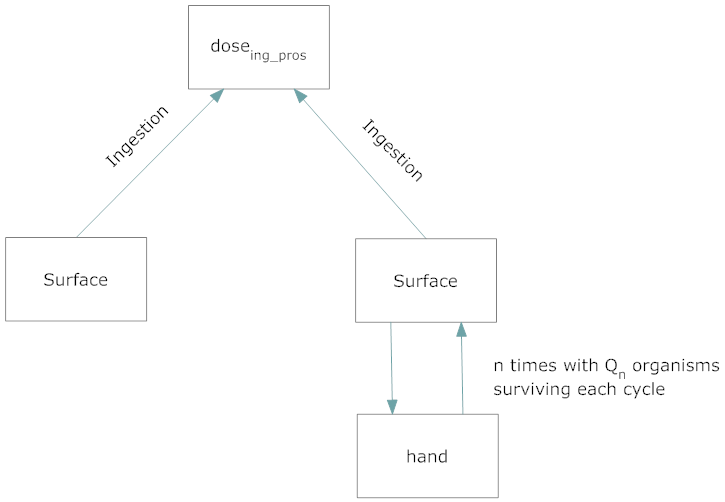


**Figure S2. Pathogen flow for estimating the ingestion dose in the prospective scenario.**

Table S1. Comparison Exposure Dose between Approximated Analytical Equation and Simulated Results (1 µm)

| Pathogen | Release scenario | Inhalation dose | | Ingestion dose | |
| --- | --- | --- | --- | --- | --- |
|  |  | Approximated analytical equation | Full numerical simulation | Approximated analytical equation | Full numerical simulation |
| *B. anthracis* | Retrospective* | 1.12×10^4^ | 1.12×10^4^ | 2.75×10^1^ | 2.37×10^1^ |
|  | Prospective* | 2.07×10^3^ | 2.10×10^3^ | 7.62×10^5^ | 7.64×10^5^ |
| *Y. pestis* | Retrospective* | 3.32×10^3^ | 3.42×10^3^ | 1.98 | 1.96 |
|  | Prospective* | 8.83×10^-1^ | 8.83×10^-1^ | 9.84×10^2^ | 9.83×10^2^ |
| *F. tularensis* | Retrospective* | 3.13×10^3^ | 3.23×10^3^ | 3.22 | 3.15 |
|  | Prospective* | 1.56 | 1.56 | 1.94×10^3^ | 1.94×10^3^ |
| *Variola major* | Retrospective* | 1.08×10^4^ | 1.08×10^4^ | 2.57×10^1^ | 2.23×10^1^ |
|  | Prospective* | 1.65×10^2^ | 1.65×10^2^ | 6.33×10^4^ | 6.32×10^4^ |
| Lassa | Retrospective* | 3.48×10^3^ | 3.58×10^3^ | 1.14 | 1.14 |
|  | Prospective* | 5.34×10^-1^ | 5.30×10^-1^ | 5.28×10^2^ | 5.27×10^2^ |

*Total release quantity is 1 million spores for both retrospective and prospective scenario. The simulation period in retrospective scenario is 8 hours, while it is one year in prospective scenario.
