# Supplementary material for: Prioritizing Risks and Uncertainties from Intentional Release of Selected Category A Pathogens
Source: PLoS One. 2012 Mar 6;7(3):e32732. doi: 10.1371/journal.pone.0032732 (PMC3295774; doi:10.1371/journal.pone.0032732)
Supplement: Information S2 — Model Inputs. (DOC) [file pone.0032732.s007.doc]

Prioritizing Risks and Uncertainties from Intentional Release of Selected Category A Pathogens

Tao Hong*^1^, Patrick L. Gurian^2^, Yin Huang^3^, and Charles N. Haas^2^

1. National Exposure Research Laboratory, EPA, Athens, GA, USA, 2. Department of Civil, Architectural, and Environmental Engineering, Drexel University, Philadelphia, PA, USA, 3. Office of Biostatistics and Epidemiology, FDA, Rockville, MD, USA

*hongtao510@gmail.com

**SUPPORTING INFORMATION**

**Supporting Information S2** Model Inputs

Table S2. Model Inputs

| Symbol | Meaning | Units | Value | | Source |
| --- | --- | --- | --- | --- | --- |
| V | Room dimensions | m^3^ | 5.6×5.6×2.5 | | Assumed a typical office |
| A_ts_ | Area-touchable surfaces | m^2^ | 5.6×5.6×0.75×0.25 | |  |
| A_tf_ | Area-tracked floor | m^2^ | 5.6×5.6×0.75×0.75 | |  |
| A_utf_ | Area-untracked floor | m^2^ | 5.6×5.6×0.25 | |  |
| A_ce_ | Area- ceiling | m^2^ | 5.6×5.6 | |  |
| A_w_ | Area- wall | m^2^ | 5.6×2.5×4 | |  |
| A_f_ | Filter area | m^2^ | 3.8×10^-2^  (2.8×10^-2^-5.6×10^-2^) | | *Q/*A = 137m/min (91-183 m/min) |
| A_n_ | Area of nasal passages | m^2^ | 0.8 | |  |
| A_h_ | Area of hand touched surface | m^2^ | 2.0×10^-3^ | | Authors' assumption |
| ACH | Air changes per hour | hr^-1^ | 4 | |  |
| Q | Discharge | m^3^/s | 0.087 | | Q = V×ACH*/*3600 (in seconds) |
| f | Proportion tracked |  | 0.75 | |  |
| Init | Amount of released pathogens | Count | 1.0×10^4^ | | Authors' assumption |
| D_dif_ | Particle diffusivity | m^2^/s | D=1μM | 2.8×10^-11^ | Estimated from |
|  |  |  | D=3μM | 8.5×10^-12^ |  |
|  |  |  | D=5μM | 5.0×10^-12^ |  |
|  |  |  | D=10μM | 2.5×10^-12^ |  |
| e_f_ | Filter efficiency |  | D=1μM | 0.098 |  |
|  |  |  | D=3μM | 0.49 |  |
|  |  |  | D=5μM | 0.74 |  |
|  |  |  | D=10μM | 0.88 |  |

Table S2. Model Inputs (Continued)

| Symbol | Meaning | Diameter | Unit | Value scale | | | | | |
| --- | --- | --- | --- | --- | --- | --- | --- | --- | --- |
|  |  |  |  | Lower bound | Source | Upper bound | Source | Input value | Source |
| e_n_ | Nasal passages particle remove efficiency | 1μM |  | 0.02 |  | 0.25 |  | 0.14 | Midpoint of range |
|  |  | 3μM |  | 0.22 |  | 0.68 |  | 0.45 |  |
|  |  | 5μM |  | 0.42 |  | 0.81 |  | 0.62 |  |
|  |  | 10μM |  | 0.62 |  | 0.91 |  | 0.77 |  |
| µ | Resuspension rate | 1μM | s^-1^ | 1.2×10^-10^ |  | 3.3×10^-8^ |  | 3.3×10^-8^ |  |
|  |  | 3μM |  | 1.7×10^-7^ |  | 1.7×10^-6^ |  | 5.3×10^-7^ |  |
|  |  | 5μM |  | 1.1×10^-6^ |  | 3.3×10^-6^ |  | 2.2×10^-7^ |  |
|  |  | 10μM |  | 8.8×10^-7^ |  | 9.4×10^-6^ |  | 1.1×10^-6^ |  |
| Inh | Breathing rate |  | m^3^/hr | 0.8 |  | 2.0 |  | 1.02 |  |
| p | Recirculation fraction |  |  | 0 |  | 1 |  | 0.75 |  |
| ρ_p_ | Density of particle |  | g/cm^3^ | 1.0 | Authors' assumption | 3.0 | Authors' assumption | 1.0 | Authors' assumption |
| k_e_ | Turbulence intensity |  | s^-1^ | 2.60×10^-2^ |  | 4.50×10^-1^ |  | 2.40×10^-1^ | Midpoint of range |

Table S2. Model Inputs (Continued)

| Symbol | Meaning (Unit) | Upper bound | Source | Lower bound | Source | Best estimate | Source |
| --- | --- | --- | --- | --- | --- | --- | --- |
| f_h-s_ | Mass transfer fraction from hand to surface during each contact | 0.338 |  | 0.010 |  | 0.174 | Middle point |
| f_s-h_ | Mass transfer fraction from surface to hand during each contact | 0.658 |  | 0.008 |  | 0.333 | Middle point |
| f_h-m_ | Mass transfer fraction from hand to mouth during each contact | 0.410 |  | 0.330 |  | 0.350 |  |
| r_h-m_ | Hand-mouth contacting rate (hr^-1^) | 8 | Authors' assumption | 5 | Authors' assumption | 8 |  |
| r_h-s_ | Hand-surface contacting rate (hr^-1^) | 6 | Authors' assumption | 3 | Authors' assumption | 6 | Authors' assumption |

Reference

1. EPA (1997) Exposure Factors Handbook: Washington, DC : Exposure Assessment Group, Office of Health and Environmental Assessment, U.S. Environmental Protection Agency.

2. Sextro RG, Lorenzetti DM, Sohn MD, Thatcher TL. Modeling the spread of anthrax in buildings; 2002; Monterey, CA. pp. 506-511.

3. Landahl H (1950) On the removal of air-borne droplets by the human respiratory tract: I. The lung. Bulletin of Mathematical Biology 12: 43-56.

4. ASHRAE (2005) ASHRAE Handbook: fundamentals. Atlanta: ASHRAE.

5. Nazaroff WW, Cass GR (1989) MASS-TRANSPORT ASPECTS OF POLLUTANT REMOVAL AT INDOOR SURFACES. Environment International 15: 567-584.

6. Schlesinger RB (1989) Deposition and Clearance of Inhaled Particles. In: McClellan RO, Henderson RF, editors. Concepts in inhalation toxicology. Washington, D.C.: Taylor&Francis. pp. 191-217.

7. Thatcher TL, Layton DW (1995) Deposition, resuspension, and penetration of particles within a residence. Atmospheric Environment 29: 1487-1497.

8. Kowalski WJ (2003) Immune Building System Technology. New York: McGraw-Hill.

9. Xu MD, Nematollahi M, Sextro RG, Gadgil AJ, Nazaroff WW (1994) Deposition of tobacco-smoke particles in a low ventilation room. Aerosol Science and Technology 20: 194-206.

10. Ansari SA, Sattar SA, Springthorpe VS, Wells GA, Tostowaryk W (1988) Rotavirus survival on human hands and transfer of infectious virus to animate and nonporous inanimate surfaces. J Clin Microbiol 26: 1513-1518.

11. Ansari SA, Springthorpe VS, Sattar SA, Rivard S, Rahman M (1991) Potential role of hands in the spread of respiratory viral infections: studies with human parainfluenza virus 3 and rhinovirus 14. J Clin Microbiol 29: 2115-2119.

12. P. Rusin SM, C. Gerba, (2002) Comparative surface-to-hand and fingertip-to-mouth transfer efficiency of gram-positive bacteria, gram-negative bacteria, and phage. Journal of Applied Microbiology 93: 585-592.

13. Nicas M, Best D (2008) A study quantifying the hand-to-face contact rate and its potential application to predicting respiratory tract infection. Journal of Occupational and Environmental Hygiene 5: 347-352.
