# Supplementary material for: Prioritizing Risks and Uncertainties from Intentional Release of Selected Category A Pathogens
Source: PLoS One. 2012 Mar 6;7(3):e32732. doi: 10.1371/journal.pone.0032732 (PMC3295774; doi:10.1371/journal.pone.0032732)
Supplement: Information S3 — Cumulative retrospective risks associated with pathogens ( F. tularensis , Variola major and Lassa) HVAC concentrations after an aerosol release. (DOC) [file pone.0032732.s008.doc]

Prioritizing Risks and Uncertainties from Intentional Release of Selected Category A Pathogens

Tao Hong*^1^, Patrick L. Gurian^2^, Yin Huang^3^, and Charles N. Haas^2^

1. National Exposure Research Laboratory, EPA, Athens, GA, USA, 2. Department of Civil, Architectural, and Environmental Engineering, Drexel University, Philadelphia, PA, USA, 3. Office of Biostatistics and Epidemiology, FDA, Rockville, MD, USA

*hongtao510@gmail.com

**SUPPORTING INFORMATION**

**Supporting Information S3** Cumulative retrospective risks associated with pathogens (*F. tularensis*, *Variola major* and Lassa) HVAC concentrations after an aerosol release

**Supporting Information S3**

**
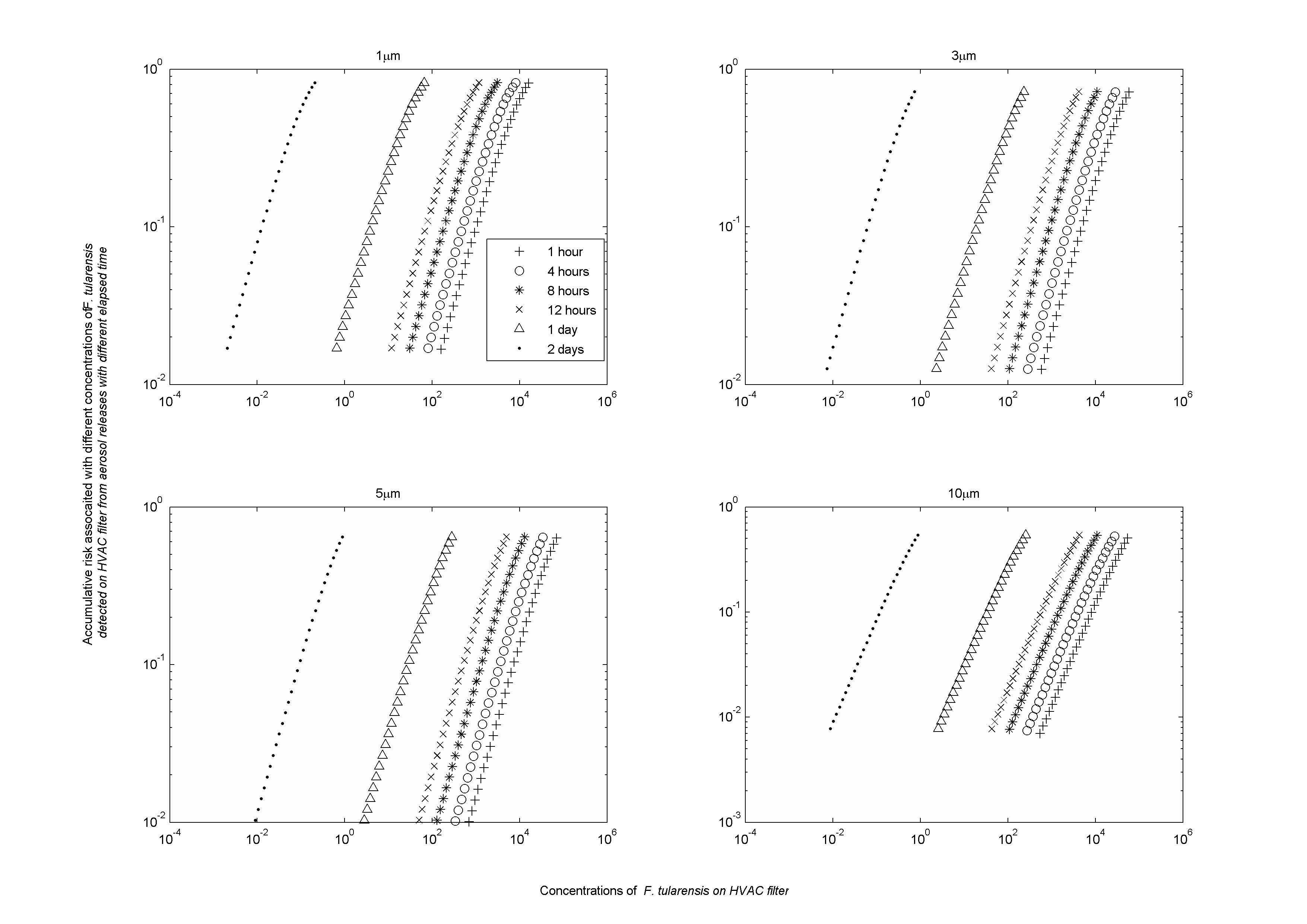
**

**Figure S3. Cumulative retrospective risks associated with *F. tularensis* HVAC concentrations after an aerosol release.**

**
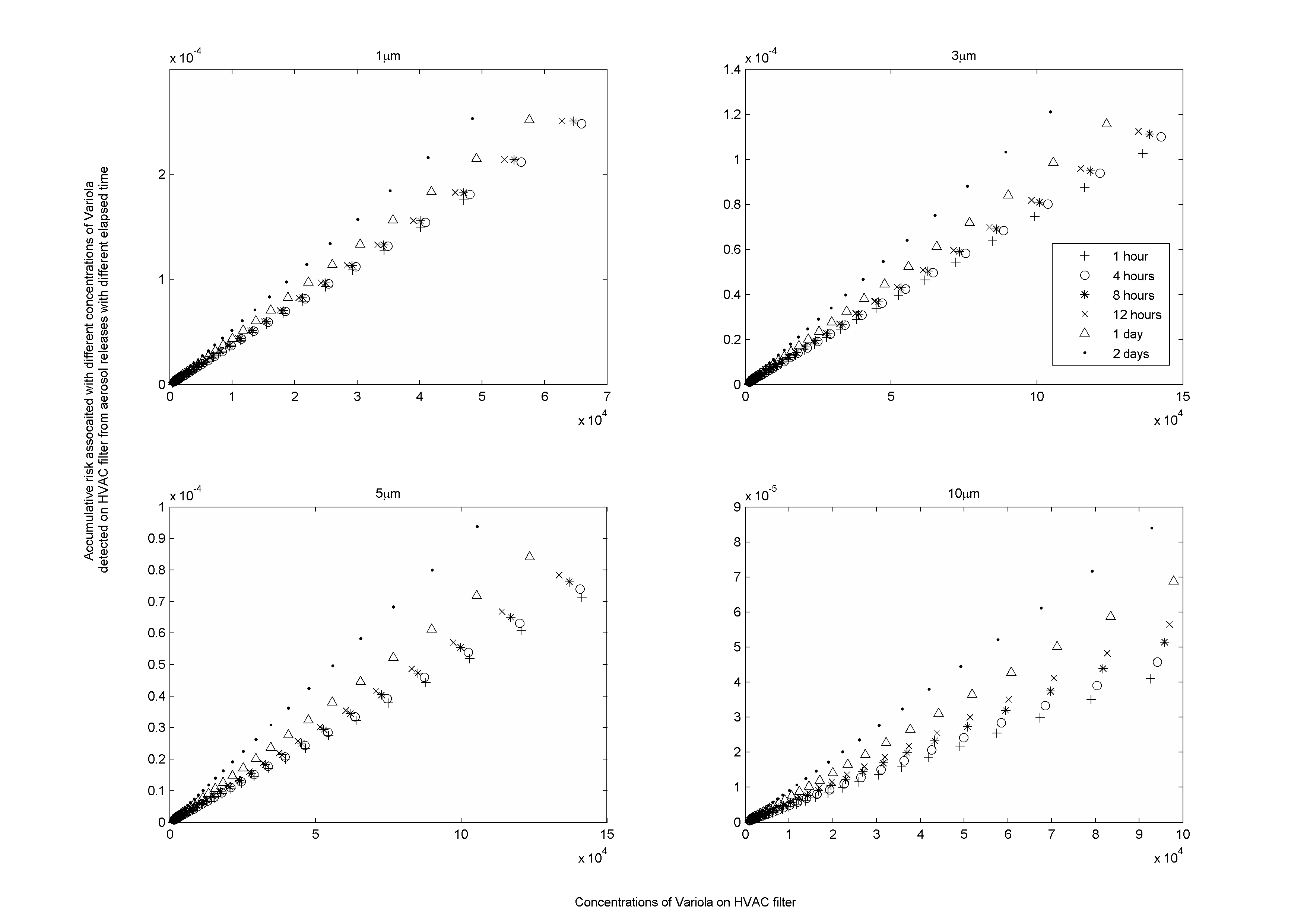
**

**Figure S4. Cumulative retrospective risks associated with *Variola major* HVAC concentrations after an aerosol release.**

**
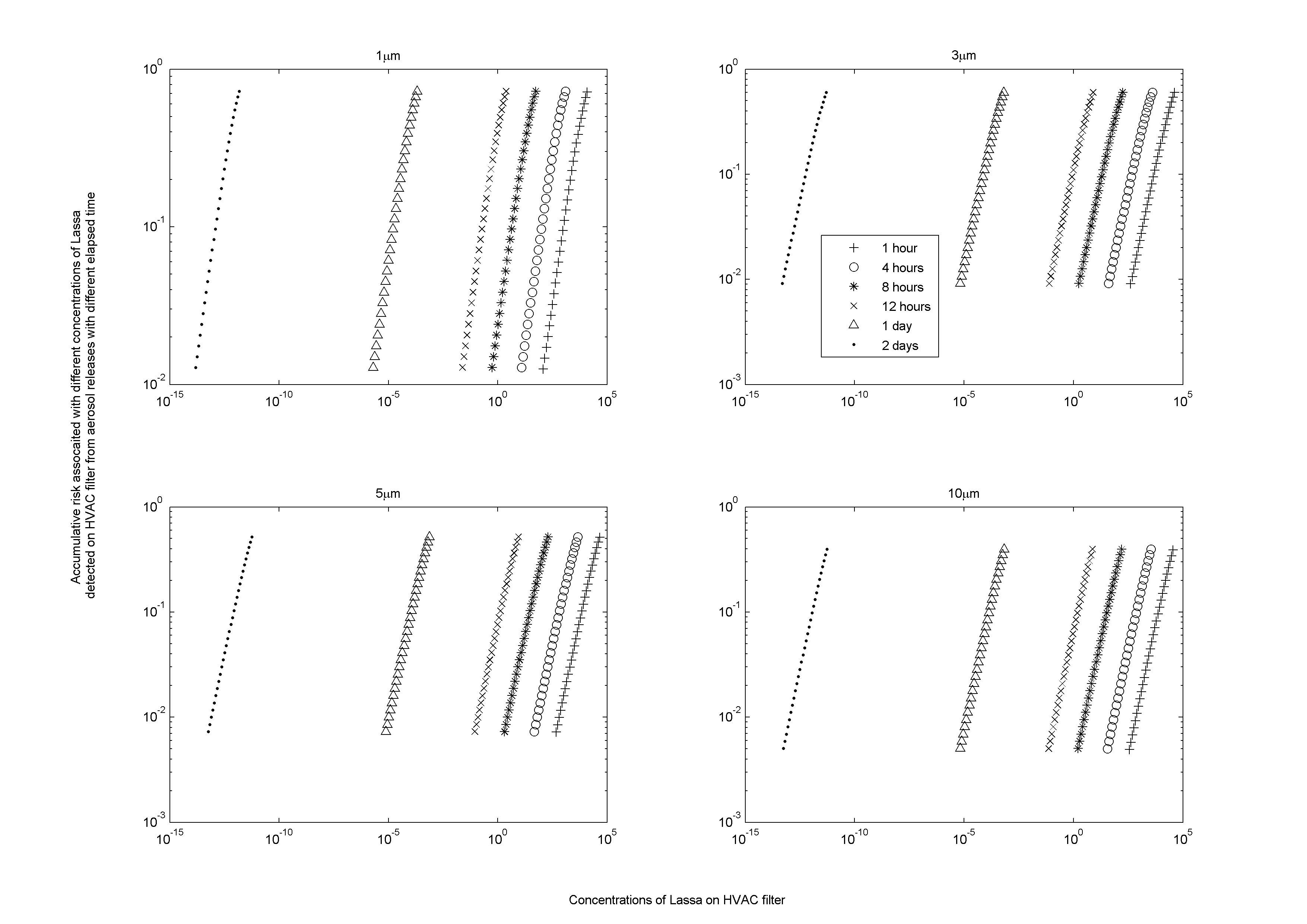
**

**Figure S5. Cumulative retrospective risks associated with Lassa HVAC concentrations after an aerosol release.**
