# Supplementary material for: Prioritizing Risks and Uncertainties from Intentional Release of Selected Category A Pathogens
Source: PLoS One. 2012 Mar 6;7(3):e32732. doi: 10.1371/journal.pone.0032732 (PMC3295774; doi:10.1371/journal.pone.0032732)
Supplement: Information S4 — Correlation coefficients between input parameters and different pathogens. (DOC) [file pone.0032732.s009.doc]

Prioritizing Risks and Uncertainties from Intentional Release of Selected Category A Pathogens

Tao Hong*^1^, Patrick L. Gurian^2^, Yin Huang^3^, and Charles N. Haas^2^

1. National Exposure Research Laboratory, EPA, Athens, GA, USA, 2. Department of Civil, Architectural, and Environmental Engineering, Drexel University, Philadelphia, PA, USA, 3. Office of Biostatistics and Epidemiology, FDA, Rockville, MD, USA

*hongtao510@gmail.com

**SUPPORTING INFORMATION**

**Supporting Information S4** Correlation coefficients between input parameters and different pathogens

**Supporting Information S4**

Table S3. Correlation coefficients for *B. anthracis*

|  |  | Retrospective scenario | | Prospective scenario | |
| --- | --- | --- | --- | --- | --- |
|  |  | Ingestion risk | Inhalation dose | Ingestion dose | Inhalation dose |
| Hand-mouth contacting rate (r_h-m_) | 1 µm | 0.0034 | -0.013 | -0.019 | -0.0093 |
|  | 3 µm | 0.0055 | -0.018 | 0.0027 | -0.0012 |
|  | 5 µm | -0.0064 | -0.019 | 0.0011 | 0.0037 |
|  | 10 µm | 0.011 | -0.021 | 0.0011 | 0.0062 |
| Hand-surface contacting rate (r_h-s_) | 1 µm | 0.071 | 0.0018 | 0.066 | -0.054 |
|  | 3 µm | 0.094 | -0.0010 | 0.086 | -0.020 |
|  | 5 µm | 0.12 | -0.0038 | 0.011 | -0.013 |
|  | 10 µm | 0.12 | -0.0096 | 0.092 | -0.011 |
| Mass transfer fraction from hand to mouth during each contact (f_h-m_) | 1 µm | -0.0084 | -0.018 | 0.00050 | -0.0093 |
|  | 3 µm | -0.00055 | -0.016 | -0.013 | -0.0012 |
|  | 5 µm | -0.0037 | -0.012 | -0.0058 | 0.0037 |
|  | 10 µm | -0.0075 | -0.0063 | 0.010 | 0.0062 |
| Mass transfer fraction from hand to surface during each contact (f_h-s_) | 1 µm | -0.0042 | 0.00070 | 0.055 | 0.047 |
|  | 3 µm | 0.0048 | 0.0050 | 0.021 | 0.020 |
|  | 5 µm | -0.0050 | 0.0075 | 0.0003 | 0.015 |
|  | 10 µm | 0.0060 | 0.0096 | 0.0085 | 0.012 |
| Mass transfer fraction from surface to hand during each contact (f_s-h_) | 1 µm | **0.21** | 0.0077 | **0.16** | -0.25 |
|  | 3 µm | **0.30** | 0.010 | **0.26** | -0.082 |
|  | 5 µm | **0.34** | 0.012 | **0.32** | -0.046 |
|  | 10 µm | **0.36** | 0.015 | **0.28** | -0.043 |
| Decay rate in this air (γ_air_) | 1 µm | 0.00030 | 0.0055 | 0.0013 | -0.0020 |
|  | 3 µm | 0.00080 | 0.0089 | 0.0013 | -0.0053 |
|  | 5 µm | 0.0075 | 0.0078 | 0.0049 | -0.0009 |
|  | 10 µm | 0.0023 | 0.0026 | -0.0021 | 0.0053 |
| Decay rate on fomite (γ_f_) | 1 µm | -0.012 | -0.017 | -0.0097 | -0.013 |
|  | 3 µm | 0.011 | -0.018 | -0.0005 | -0.00080 |
|  | 5 µm | 0.0022 | -0.018 | 0.0002 | 0.0022 |
|  | 10 µm | -0.00090 | -0.018 | -0.0070 | 0.0031 |
| Dose-response coefficient | 1 µm | **0.66** | 0.058 | **0.87** | 0.027 |
|  | 3 µm | **0.72** | 0.088 | **0.73** | 0.053 |
|  | 5 µm | **0.76** | 0.11 | **0.77** | 0.075 |
|  | 10 µm | **0.76** | 0.14 | **0.63** | 0.012 |

Table S3. Correlation coefficients for *B. anthracis* (continued)

|  |  | Retrospective scenario | | Prospective scenario | |
| --- | --- | --- | --- | --- | --- |
|  |  | Ingestion dose | Inhalation dose | Ingestion dose | Inhalation dose |
| Breathing rate (Inh) | 1 µm | 0.0065 | **0.27** | 0.021 | **0.14** |
|  | 3 µm | -0.0065 | **0.40** | 0.017 | **0.25** |
|  | 5 µm | -0.00040 | **0.53** | 0.0067 | **0.34** |
|  | 10 µm | 0.0021 | **0.65** | -0.0026 | **0.53** |
| Nasal passages particle remove efficiency (e_n_) | 1 µm | -0.0030 | -0.0093 | -0.0064 | -0.0038 |
|  | 3 µm | 0.0028 | 0.0020 | 0.0099 | -0.012 |
|  | 5 µm | 0.0036 | -0.017 | 0.00070 | -0.0010 |
|  | 10 µm | 0.0029 | 0.0083 | -0.00090 | 0.0078 |
| Air change rate (ACH) | 1 µm | **-0.18** | **-0.68** | -0.0038 | **-0.44** |
|  | 3 µm | **-0.21** | **-0.72** | -0.029 | **-0.70** |
|  | 5 µm | **-0.16** | **-0.62** | -0.052 | **-0.75** |
|  | 10 µm | **-0.081** | **-0.31** | -0.052 | **-0.63** |
| Resuspension rate (µ_2_) | 1 µm | -0.0040 | 0.00060 | **-0.047** | **0.32** |
|  | 3 µm | 0.0070 | -0.0012 | **-0.26** | **0.088** |
|  | 5 µm | -0.012 | 0.0052 | **-0.18** | **0.022** |
|  | 10 µm | -0.0068 | 0.089 | **-0.29** | **0.058** |
| Turbulence intensity (k_e_) | 1 µm | 0.013 | 0.0084 | -0.0024 | -0.0040 |
|  | 3 µm | 0.0023 | 0.012 | -0.013 | 0.00050 |
|  | 5 µm | 0.011 | 0.012 | -0.0059 | 0.0031 |
|  | 10 µm | 0.0077 | 0.0067 | -0.0067 | 0.0043 |
| Density of the particle (ρ_p_) | 1 µm | 0.10 | **-0.052** | -0.0049 | -0.035 |
|  | 3 µm | 0.080 | **-0.24** | 0.018 | -0.096 |
|  | 5 µm | 0.075 | **-0.40** | 0.014 | -0.17 |
|  | 10 µm | 0.030 | **-0.61** | 0.011 | -0.40 |

**Table S4. Correlation coefficients for *Y. pestis***

|  |  | Retrospective scenario | | Prospective scenario | |
| --- | --- | --- | --- | --- | --- |
|  |  | Ingestion dose | Inhalation dose | Ingestion dose | Inhalation dose |
| Hand-mouth contacting rate (r_h-m_) | 1 µm | 0.032 | 0.0033 | 0.0071 | -0.011 |
|  | 3 µm | 0.035 | 0.0087 | 0.0079 | -0.015 |
|  | 5 µm | 0.038 | 0.012 | 0.0085 | -0.0069 |
|  | 10 µm | 0.042 | 0.015 | 0.010 | -0.013 |
| Hand-surface contacting rate (r_h-s_) | 1 µm | 0.13 | -0.012 | **0.071** | -0.014 |
|  | 3 µm | 0.14 | -0.014 | **0.074** | -0.017 |
|  | 5 µm | 0.14 | -0.017 | **0.076** | -0.011 |
|  | 10 µm | 0.14 | -0.022 | **0.079** | -0.0067 |
| Mass transfer fraction from hand to mouth during each contact (f_h-m_) | 1 µm | 0.029 | -0.034 | 0.037 | 0.026 |
|  | 3 µm | 0.028 | -0.035 | 0.036 | 0.036 |
|  | 5 µm | 0.029 | -0.034 | 0.038 | 0.025 |
|  | 10 µm | 0.030 | -0.029 | 0.036 | 0.026 |
| Mass transfer fraction from hand to surface during each contact (f_h-s_) | 1 µm | -0.11 | -0.0019 | -0.059 | 0.027 |
|  | 3 µm | -0.12 | -0.0024 | -0.060 | 0.0053 |
|  | 5 µm | -0.12 | -0.0028 | -0.062 | 0.023 |
|  | 10 µm | -0.12 | -0.0022 | -0.065 | 0.013 |
| Mass transfer fraction from surface to hand during each contact (f_s-h_) | 1 µm | **0.47** | 0.014 | **0.34** | -0.0088 |
|  | 3 µm | **0.48** | 0.020 | **0.35** | 0.0026 |
|  | 5 µm | **0.49** | 0.021 | **0.36** | -0.015 |
|  | 10 µm | **0.51** | 0.018 | **0.38** | -0.0023 |
| Decay rate in this air (γ_air_) | 1 µm | -0.10 | -0.30 | -0.0057 | -0.079 |
|  | 3 µm | -0.082 | -0.24 | -0.0066 | -0.058 |
|  | 5 µm | -0.066 | -0.18 | -0.0059 | -0.066 |
|  | 10 µm | -0.042 | -0.083 | -0.0051 | -0.042 |
| Decay rate on fomite (γ_f_) | 1 µm | **-0.56** | -0.0029 | **-0.53** | **-0.51** |
|  | 3 µm | **-0.57** | 0.0037 | **-0.54** | **-0.56** |
|  | 5 µm | **-0.59** | 0.0048 | **-0.55** | **-0.63** |
|  | 10 µm | **-0.61** | -0.0072 | **-0.56** | **-0.57** |
| Dose-response coefficient | 1 µm | 0.0058 | 0.053 | 0.032 | 0.032 |
|  | 3 µm | 0.0039 | 0.056 | 0.032 | 0.035 |
|  | 5 µm | 0.0039 | 0.058 | 0.032 | 0.028 |
|  | 10 µm | 0.0055 | 0.059 | 0.030 | 0.038 |

**Table S4. Correlation coefficients for *Y. pestis* (continued)**

|  |  | Retrospective scenario | | Prospective scenario | |
| --- | --- | --- | --- | --- | --- |
|  |  | Ingestion dose | Inhalation dose | Ingestion dose | Inhalation dose |
| Breathing rate (Inh) | 1 µm | 0.0072 | **0.73** | -0.014 | **0.15** |
|  | 3 µm | 0.0048 | **0.76** | -0.014 | **0.16** |
|  | 5 µm | 0.0028 | **0.78** | -0.014 | **0.18** |
|  | 10 µm | -0.00091 | **0.76** | -0.015 | **0.16** |
| Nasal passages particle remove efficiency (e_n_) | 1 µm | -0.015 | 0.011 | -0.022 | -0.012 |
|  | 3 µm | 0.0029 | -0.014 | -0.011 | -0.0053 |
|  | 5 µm | 0.0019 | 0.0060 | 0.0088 | -0.0048 |
|  | 10 µm | 0.0087 | -0.0079 | -0.00028 | -0.0015 |
| Air change rate (ACH) | 1 µm | -0.11 | **-0.44** | 0.0036 | -0.080 |
|  | 3 µm | -0.13 | **-0.52** | 0.0034 | -0.11 |
|  | 5 µm | -0.12 | **-0.48** | 0.0035 | -0.11 |
|  | 10 µm | -0.070 | **-0.28** | 0.0023 | -0.057 |
| Resuspension rate (µ_2_) | 1 µm | -0.0018 | 0.026 | -0.0048 | **0.35** |
|  | 3 µm | -0.028 | 0.018 | -0.0013 | **0.31** |
|  | 5 µm | -0.00099 | 0.023 | -0.016 | **0.21** |
|  | 10 µm | -0.00023 | 0.019 | -0.019 | **0.33** |
| Turbulence intensity (k_e_) | 1 µm | 0.0040 | -0.010 | 0.0018 | 0.015 |
|  | 3 µm | 0.0037 | -0.0064 | 0.0020 | 0.0074 |
|  | 5 µm | 0.0037 | -0.0039 | 0.0023 | 0.013 |
|  | 10 µm | 0.0037 | -0.0015 | 0.0019 | 0.010 |
| Density of the particle (ρ_p_) | 1 µm | **0.24** | **-0.026** | -0.0083 | -0.0058 |
|  | 3 µm | **0.22** | **-0.15** | -0.0076 | -0.033 |
|  | 5 µm | **0.19** | **-0.30** | -0.0071 | -0.070 |
|  | 10 µm | **0.12** | **-0.54** | -0.0085 | -0.12 |

**Table S5. Correlation coefficients for *F. tularensis***

|  |  | Retrospective scenario | | Prospective scenario | |
| --- | --- | --- | --- | --- | --- |
|  |  | Ingestion dose | Inhalation dose | Ingestion dose | Inhalation dose |
| Hand-mouth contacting rate (r_h-m_) | 1 µm | 0.033 | 0.016 | 0.011 | 0.0079 |
|  | 3 µm | 0.033 | 0.017 | 0.011 | 0.0071 |
|  | 5 µm | 0.032 | 0.017 | 0.013 | -0.0044 |
|  | 10 µm | 0.033 | 0.019 | 0.017 | -0.0082 |
| Hand-surface contacting rate (r_h-s_) | 1 µm | 0.14 | 0.0020 | 0.12 | 0.020 |
|  | 3 µm | 0.17 | 0.0024 | 0.12 | 0.017 |
|  | 5 µm | 0.18 | 0.00099 | 0.13 | 0.021 |
|  | 10 µm | 0.20 | -0.0019 | 0.13 | 0.023 |
| Mass transfer fraction from hand to mouth during each contact (f_h-m_) | 1 µm | 0.0069 | 0.0043 | 0.033 | 0.0091 |
|  | 3 µm | 0.011 | 0.0094 | 0.034 | 0.0080 |
|  | 5 µm | 0.015 | 0.013 | 0.035 | 0.0067 |
|  | 10 µm | 0.020 | 0.017 | 0.038 | 0.010 |
| Mass transfer fraction from hand to surface during each contact (f_h-s_) | 1 µm | -0.12 | -0.0077 | -0.085 | 0.0063 |
|  | 3 µm | -0.14 | -0.0037 | -0.090 | 0.00034 |
|  | 5 µm | -0.15 | 0.0023 | -0.096 | 0.014 |
|  | 10 µm | -0.17 | 0.017 | -0.10 | 0.0060 |
| Mass transfer fraction from surface to hand during each contact (f_s-h_) | 1 µm | **0.44** | -0.012 | **0.41** | 0.0031 |
|  | 3 µm | **0.52** | -0.016 | **0.42** | -0.0039 |
|  | 5 µm | **0.58** | -0.018 | **0.44** | -0.0030 |
|  | 10 µm | **0.67** | -0.023 | **0.47** | -0.00091 |
| Decay rate in this air (γ_air_) | 1 µm | **-0.43** | **-0.75** | 0.0055 | **-0.26** |
|  | 3 µm | **-0.40** | **-0.73** | 0.0050 | **-0.24** |
|  | 5 µm | **-0.35** | **-0.67** | 0.0035 | **-0.22** |
|  | 10 µm | **-0.23** | **-0.46** | -0.0036 | **-0.14** |
| Decay rate on fomite (γ_f_) | 1 µm | **-0.33** | **-0.18** | **-0.65** | **-0.42** |
|  | 3 µm | **-0.39** | **-0.21** | **-0.65** | **-0.50** |
|  | 5 µm | **-0.43** | **-0.26** | **-0.64** | **-0.56** |
|  | 10 µm | **-0.49** | **-0.42** | **-0.64** | **-0.59** |
| Dose-response coefficient | 1 µm | 0.015 | 0.0049 | 0.021 | 0.0015 |
|  | 3 µm | 0.017 | 0.0091 | 0.022 | -0.00098 |
|  | 5 µm | 0.017 | 0.0097 | 0.022 | -0.000097 |
|  | 10 µm | 0.016 | 0.0061 | 0.020 | 0.013 |

**Table S5. Correlation coefficients for *F.tularensis* (continued)**

|  |  | Retrospective scenario | | Prospective scenario | |
| --- | --- | --- | --- | --- | --- |
|  |  | Ingestion dose | Inhalation dose | Ingestion dose | Inhalation dose |
| Breathing rate (Inh) | 1 µm | -0.023 | **0.35** | -0.011 | 0.12 |
|  | 3 µm | -0.029 | **0.45** | -0.013 | 0.14 |
|  | 5 µm | -0.033 | **0.54** | -0.015 | 0.17 |
|  | 10 µm | -0.038 | **0.69** | -0.018 | 0.20 |
| Nasal passages particle remove efficiency (e_n_) | 1 µm | -0.024 | -0.0042 | 0.011 | 0.00061 |
|  | 3 µm | 0.015 | 0.00042 | 0.0077 | -0.018 |
|  | 5 µm | 0.00056 | 0.0051 | 0.010 | -0.013 |
|  | 10 µm | 0.0072 | -0.00095 | 0.0066 | 0.012 |
| Air change rate (ACH) | 1 µm | -0.13 | -0.23 | 0.0025 | -0.087 |
|  | 3 µm | -0.16 | -0.30 | 0.0026 | -0.10 |
|  | 5 µm | -0.15 | -0.31 | 0.0036 | -0.11 |
|  | 10 µm | -0.089 | -0.22 | 0.0048 | -0.073 |
| Resuspension rate (µ_2_) | 1 µm | 0.0029 | -0.0027 | 0.0053 | **0.26** |
|  | 3 µm | -0.0079 | 0.015 | -0.0075 | **0.26** |
|  | 5 µm | -0.016 | -0.011 | -0.020 | **0.18** |
|  | 10 µm | -0.0076 | 0.038 | -0.060 | **0.33** |
| Turbulence intensity (k_e_) | 1 µm | 0.0032 | 0.018 | **0.020** | 0.018 |
|  | 3 µm | -0.0020 | 0.013 | **0.020** | 0.0078 |
|  | 5 µm | -0.0056 | 0.010 | **0.020** | 0.00072 |
|  | 10 µm | -0.011 | 0.0089 | **0.021** | -0.011 |
| Density of the particle (ρ_p_) | 1 µm | 0.22 | -0.0066 | -0.019 | -0.014 |
|  | 3 µm | 0.23 | -0.077 | -0.019 | -0.040 |
|  | 5 µm | 0.21 | -0.18 | -0.018 | -0.067 |
|  | 10 µm | 0.15 | -0.44 | -0.015 | -0.13 |

**Table S6. Correlation coefficients for *Variola major***

|  |  | Retrospective scenario | | Prospective scenario | |
| --- | --- | --- | --- | --- | --- |
|  |  | Ingestion dose | Inhalation dose | Ingestion dose | Inhalation dose |
| Hand-mouth contacting rate (r_h-m_) | 1 µm | 0.070 | 0.040 | 0.057 | -0.018 |
|  | 3 µm | 0.080 | 0.048 | 0.062 | -0.013 |
|  | 5 µm | 0.081 | 0.044 | 0.056 | -0.0078 |
|  | 10 µm | 0.075 | 0.033 | 0.061 | -0.0065 |
| Hand-surface contacting rate (r_h-s_) | 1 µm | 0.10 | -0.0070 | 0.19 | 0.0013 |
|  | 3 µm | 0.12 | -0.0092 | 0.19 | 0.00018 |
|  | 5 µm | 0.14 | -0.0060 | 0.19 | -0.0045 |
|  | 10 µm | 0.16 | 0.0036 | 0.17 | -0.0011 |
| Mass transfer fraction from hand to mouth during each contact (f_h-m_) | 1 µm | 0.021 | 0.013 | 0.016 | 0.0092 |
|  | 3 µm | 0.016 | 0.0036 | 0.019 | 0.0047 |
|  | 5 µm | 0.013 | -0.0039 | 0.021 | 0.0088 |
|  | 10 µm | 0.011 | -0.013 | 0.025 | 0.0016 |
| Mass transfer fraction from hand to surface during each contact (f_h-s_) | 1 µm | -0.11 | 0.0027 | -0.16 | 0.022 |
|  | 3 µm | -0.13 | 0.0025 | -0.17 | 0.030 |
|  | 5 µm | -0.15 | 0.0039 | -0.17 | 0.029 |
|  | 10 µm | -0.16 | 0.0044 | -0.15 | 0.027 |
| Mass transfer fraction from surface to hand during each contact (f_s-h_) | 1 µm | **0.45** | -0.013 | **0.67** | -0.033 |
|  | 3 µm | **0.54** | -0.012 | **0.67** | -0.017 |
|  | 5 µm | **0.61** | -0.011 | **0.67** | -0.011 |
|  | 10 µm | **0.67** | -0.0064 | **0.60** | 0.00058 |
| Decay rate in this air (γ_air_) | 1 µm | -0.023 | -0.055 | -0.010 | -0.19 |
|  | 3 µm | -0.014 | -0.032 | -0.015 | -0.14 |
|  | 5 µm | -0.0090 | -0.024 | -0.0066 | -0.11 |
|  | 10 µm | -0.0043 | -0.017 | -0.013 | -0.081 |
| Decay rate on fomite (γ_f_) | 1 µm | 0.0045 | 0.0030 | -0.059 | -0.11 |
|  | 3 µm | -0.0021 | -0.0025 | -0.017 | -0.15 |
|  | 5 µm | -0.0072 | -0.0047 | -0.014 | -0.15 |
|  | 10 µm | -0.012 | -0.0060 | -0.00080 | -0.12 |
| Dose-response coefficient | 1 µm | **0.38** | **0.44** | **0.57** | **0.30** |
|  | 3 µm | **0.45** | **0.57** | **0.56** | **0.45** |
|  | 5 µm | **0.50** | **0.67** | **0.56** | **0.57** |
|  | 10 µm | **0.54** | **0.73** | **0.51** | **0.61** |

**Table S6 Correlation coefficients for *Variola major* (continued)**

|  |  | Retrospective scenario | | Prospective scenario | |
| --- | --- | --- | --- | --- | --- |
|  |  | Ingestion dose | Inhalation dose | Ingestion dose | Inhalation dose |
| Breathing rate (Inh) | 1 µm | -0.0043 | **0.25** | 0.010 | 0.18 |
|  | 3 µm | -0.00019 | **0.32** | 0.0038 | 0.27 |
|  | 5 µm | 0.00032 | **0.37** | 0.0099 | 0.34 |
|  | 10 µm | -0.00060 | **0.40** | 0.015 | 0.36 |
| Nasal passages particle remove efficiency (e_n_) | 1 µm | -0.015 | -0.077 | -0.0043 | -0.025 |
|  | 3 µm | -0.048 | -0.024 | 0.0057 | 0.018 |
|  | 5 µm | -0.015 | 0.0052 | 0.013 | -0.00094 |
|  | 10 µm | -0.0092 | -0.0059 | -0.011 | -0.0064 |
| Air change rate (ACH) | 1 µm | **-0.37** | **-0.54** | 0.018 | **-0.41** |
|  | 3 µm | **-0.36** | **-0.53** | 0.0042 | **-0.45** |
|  | 5 µm | **-0.27** | **-0.42** | -0.0092 | **-0.45** |
|  | 10 µm | **-0.11** | **-0.19** | -0.028 | **-0.25** |
| Resuspension rate (µ_2_) | 1 µm | 0.0031 | -0.0038 | **-0.46** | **0.36** |
|  | 3 µm | 0.0045 | 0.0069 | **-0.15** | **0.39** |
|  | 5 µm | -0.0041 | 0.011 | **-0.16** | **0.25** |
|  | 10 µm | -0.013 | 0.039 | **-0.35** | **0.39** |
| Turbulence intensity (k_e_) | 1 µm | 0.0043 | 0.010 | -0.022 | -0.015 |
|  | 3 µm | 0.0096 | 0.016 | -0.018 | -0.017 |
|  | 5 µm | 0.0077 | 0.013 | -0.020 | -0.012 |
|  | 10 µm | 0.0028 | 0.055 | -0.024 | -0.0089 |
| Density of the particle (ρ_p_) | 1 µm | 0.19 | -0.069 | -0.0065 | -0.039 |
|  | 3 µm | 0.14 | -0.20 | -0.0022 | -0.14 |
|  | 5 µm | 0.099 | -0.29 | 0.0025 | -0.21 |
|  | 10 µm | 0.031 | -0.40 | 0.018 | -0.31 |

**Table S7 Correlation coefficients for Lassa**

|  |  | Retrospective scenario | | Prospective scenario | |
| --- | --- | --- | --- | --- | --- |
|  |  | Ingestion dose | Inhalation dose | Ingestion dose | Inhalation dose |
| Hand-mouth contacting rate (r_h-m_) | 1 µm | 0.014 | 0.0017 | 0.033 | 0.0088 |
|  | 3 µm | 0.017 | 0.0035 | 0.033 | 0.022 |
|  | 5 µm | 0.020 | 0.0059 | 0.033 | 0.021 |
|  | 10 µm | 0.024 | 0.0095 | 0.033 | 0.019 |
| Hand-surface contacting rate (r_h-s_) | 1 µm | 0.098 | -0.022 | -0.055 | -0.0023 |
|  | 3 µm | 0.10 | -0.024 | -0.055 | -0.0031 |
|  | 5 µm | 0.11 | -0.027 | -0.055 | -0.0081 |
|  | 10 µm | 0.11 | -0.031 | -0.055 | -0.0083 |
| Mass transfer fraction from hand to mouth during each contact (f_h-m_) | 1 µm | 0.019 | -0.0038 | 0.033 | 0.0088 |
|  | 3 µm | 0.023 | -0.0042 | 0.033 | 0.022 |
|  | 5 µm | 0.024 | -0.0050 | 0.033 | 0.021 |
|  | 10 µm | 0.025 | -0.0079 | 0.033 | 0.019 |
| Mass transfer fraction from hand to surface during each contact (f_h-s_) | 1 µm | -0.074 | 0.011 | -0.055 | -0.0023 |
|  | 3 µm | -0.083 | 0.0077 | -0.055 | -0.0031 |
|  | 5 µm | -0.090 | 0.0045 | -0.055 | -0.0081 |
|  | 10 µm | -0.098 | 0.0018 | -0.055 | -0.0083 |
| Mass transfer fraction from surface to hand during each contact (f_s-h_) | 1 µm | **0.40** | -0.018 | **0.51** | 0.0027 |
|  | 3 µm | **0.42** | -0.016 | **0.51** | -0.0058 |
|  | 5 µm | **0.44** | -0.013 | **0.51** | -0.0015 |
|  | 10 µm | **0.47** | -0.0086 | **0.51** | -0.012 |
| Decay rate in this air (γ_air_) | 1 µm | **-0.21** | **-0.31** | -0.025 | **-0.24** |
|  | 3 µm | **-0.16** | **-0.24** | -0.025 | **-0.18** |
|  | 5 µm | **-0.12** | **-0.18** | -0.025 | **-0.17** |
|  | 10 µm | **-0.057** | **-0.082** | -0.025 | **-0.089** |
| Decay rate on fomite (γ_f_) | 1 µm | -0.092 | -0.017 | -0.093 | -0.057 |
|  | 3 µm | -0.099 | -0.018 | -0.093 | -0.057 |
|  | 5 µm | -0.11 | -0.020 | -0.092 | -0.079 |
|  | 10 µm | -0.11 | -0.025 | -0.091 | -0.078 |
| Dose-response coefficient | 1 µm | **0.60** | **0.69** | **0.72** | **0.61** |
|  | 3 µm | **0.64** | **0.75** | **0.72** | **0.73** |
|  | 5 µm | **0.67** | **0.80** | **0.72** | **0.80** |
|  | 10 µm | **0.70** | **0.87** | **0.72** | **0.73** |

**Table S7 Correlation coefficients for Lassa (continued)**

|  |  | Retrospective scenario | | Prospective scenario | |
| --- | --- | --- | --- | --- | --- |
|  |  | Ingestion dose | Inhalation dose | Ingestion dose | Inhalation dose |
| Breathing rate (Inh) | 1 µm | 0.0066 | **0.26** | 0.011 | **0.17** |
|  | 3 µm | 0.0093 | **0.27** | 0.011 | **0.19** |
|  | 5 µm | 0.011 | **0.28** | 0.011 | **0.23** |
|  | 10 µm | 0.012 | **0.28** | 0.011 | **0.20** |
| Nasal passages particle remove efficiency (e_n_) | 1 µm | 0.016 | 0.030 | 0.0022 | 0.011 |
|  | 3 µm | 0.0040 | -0.012 | -0.0033 | 0.0050 |
|  | 5 µm | -0.0049 | 0.0014 | 0.016 | 0.011 |
|  | 10 µm | 0.0029 | 0.018 | 0.010 | 0.0022 |
| Air change rate (ACH) | 1 µm | -0.13 | -0.20 | -0.0030 | -0.15 |
|  | 3 µm | -0.14 | -0.22 | -0.0031 | -0.16 |
|  | 5 µm | -0.13 | -0.19 | -0.0031 | -0.17 |
|  | 10 µm | -0.070 | -0.098 | -0.0031 | -0.085 |
| Resuspension rate (µ_2_) | 1 µm | 0.0065 | 0.0084 | **0.40** | 0.021 |
|  | 3 µm | 0.022 | 0.016 | **0.38** | 0.026 |
|  | 5 µm | 0.0090 | 0.0030 | **0.26** | 0.0042 |
|  | 10 µm | 0.011 | 0.00046 | **0.37** | -0.021 |
| Turbulence intensity (k_e_) | 1 µm | 0.0054 | -0.0056 | -0.0013 | 0.0067 |
|  | 3 µm | 0.0069 | -0.0041 | -0.0013 | -0.0021 |
|  | 5 µm | 0.0080 | -0.0023 | -0.0014 | 0.0063 |
|  | 10 µm | 0.0098 | 0.00054 | -0.0013 | -0.0033 |
| Density of the particle (ρ_p_) | 1 µm | 0.19 | -0.0056 | 0.0085 | 0.011 |
|  | 3 µm | 0.17 | -0.050 | 0.0085 | -0.033 |
|  | 5 µm | 0.15 | -0.11 | 0.0086 | -0.090 |
|  | 10 µm | 0.087 | -0.19 | 0.0088 | -0.16 |
